# Methods, strategies, and incentives to increase response to mental health surveys among adolescents: a systematic review

**DOI:** 10.1186/s12874-023-02096-z

**Published:** 2023-11-16

**Authors:** Julia Bidonde, Jose F. Meneses-Echavez, Elisabet Hafstad, Geir Scott Brunborg, Lasse Bang

**Affiliations:** 1 Division of Health Services, Norwegian Institute of Public Health, Oslo, Norway; 2 Facultad de Cultura Física, Deporte, y Recreación, Universidad Santo Tomás, Bogotá, Colombia; 3 Department of Child Health and Development, Norwegian Institute of Public Health, Oslo, Norway; 4 Department of Clinical Neuroscience, Karolinska Institutet, Stockholm, Sweden

**Keywords:** Adolescents, Mental health, Surveys and questionnaires, Interviews, Systematic review

## Abstract

**Background:**

This systematic review aimed to identify effective methods to increase adolescents’ response to surveys about mental health and substance use, to improve the quality of survey information.

**Methods:**

We followed a protocol and searched for studies that compared different survey delivery modes to adolescents. Eligible studies reported response rates, mental health score variation per survey mode and participant variations in mental health scores. We searched CENTRAL, PsycINFO, MEDLINE and Scopus in May 2022, and conducted citation searches in June 2022. Two reviewers independently undertook study selection, data extraction, and risk of bias assessments. Following the assessment of heterogeneity, some studies were pooled using meta-analysis.

**Results:**

Fifteen studies were identified, reporting six comparisons related to survey methods and strategies. Results indicate that response rates do not differ between survey modes (e.g., web versus paper-and-pencil) delivered in classroom settings. However, web surveys may yield higher response rates outside classroom settings. The largest effects on response rates were achieved using unconditional monetary incentives and obtaining passive parental consent. Survey mode influenced mental health scores in certain comparisons.

**Conclusions:**

Despite the mixed quality of the studies, the low volume for some comparisons and the limit to studies in high income countries, several effective methods and strategies to improve adolescents’ response rates to mental health surveys were identified.

**Supplementary Information:**

The online version contains supplementary material available at 10.1186/s12874-023-02096-z.

## Background

Globally, one in seven adolescents (aged 10–19 years) experiences a mental disorder, accounting for 13% of the health burden in this age group [1]. The Global Burden of Diseases Study reports that anxiety disorders, depressive disorders and self-harm are among the top ten leading causes of adolescent health loss [2]. Understanding the magnitude and determinants of mental health problems among adolescents may inform initiatives to improve their health.

Survey research methods are often used to investigate the prevalence and incidence of mental health problems and associated risk factors and outcomes [3–5]. Prevalence estimates are based on responses from a sample of the target population. A major priority is to ensure that invited adolescents participate in the survey. In survey research, the response rate (also known as completion rate or return rate) is a crucial metric that indicates the proportion of individuals who participated in the survey divided by the total number of people in the selected sample. Non-response reduces the sample size and statistical precision of the estimates and may also induce non-response bias [6, 7]. Consequently, survey response rate is often considered an indicator of the quality and representativeness of the obtained data [6, 8].

Non-response is a particular concern in surveys of adolescents as this age-group is hard to reach and motivate to participate in research. Furthermore, response rates for health-related surveys are declining [3, 5]. For example, the response rate for a repeated household survey conducted in the US dropped by 35 percentage points between 1971 and 2017 [9]. Similarly, response rates for the National Health and Nutrition Examination Survey (NHANES) dropped by 15 percentage points from 2011/2012 to 2017/2018 [10]. There is an increasing need for surveys to be designed and administered in ways that maximise response rates. Multiple published reviews [11–13] provide evidence of methods and strategies to increase response rates (primarily among adults). These point to several factors associated with increased response rate, including the use of monetary incentives, short questionnaires and notifying participants before sending questionnaires. However, none of these focuses specifically on adolescent samples. Survey characteristics may impact response rates differently in adult and adolescent samples due to age-specific attitudes. For example, adolescents may find web surveys more acceptable and appealing than telephone or postal surveys. Attitudes towards incentives or the topic of surveys (e.g., mental health) may also differ between adults and adolescents. Furthermore, surveys of adolescents are often conducted in class-room settings which exerts a strong contextual influence on response rates. Such contextual factors may moderate the effect of methods and strategies that have been shown to influence response rates among adults.

Features that boost response rates may also influence the mental health outcomes obtained. For example, web-based surveys may improve response rates due to the relative ease of participation when compared with in-person surveys. But they may also impact mental health scores, leading to higher or lower estimates of the prevalence of mental health problems. For example, this can occur because of reluctance to disclose mental health problems to an interviewer, or because web-surveys elicit careless responses. Some studies suggest that mental health indicators differ according to the mode of data collection [14–16]. Consequently, we need to know which strategies and methods improve adolescents' response rates to mental health surveys and how these might impact mental health scores.

Many factors may positively affect response rates in surveys, including how potential participants are approached and informed about the survey (e.g., pre-notifications), incentives (e.g., financial compensation), data collection mode (e.g., web-based vs. paper-and-pencil), survey measure composition and design (e.g., questionnaire length), using follow-up reminders, and practical issues such as time and location [11, 16].

This review aims to identify effective methods and strategies to increase adolescents’ response rates (which may improve the quality of information gathered) to surveys that include questions about mental health, alcohol, and substance use. It also explores how different modes of survey delivery may impact on mental health scores. To accommodate recent trends in technological improvements and attitudes we focus on studies that have been published after 2007. By choosing 2007 we covered advances in technology since the development of the smart phone, and the literature after a previous review [13] whose search was completed in 2008. Furthermore, to provide the best quality evidence we focus on studies with randomised controlled designs.

## Methods

This systematic review used the Cochrane approach to methodology reviews [17]. The full protocol was peer reviewed and is publicly available [18], but was not registered. The review is reported according to the PRISMA guidelines [19]. Amendments to the protocol can be found in Additional file 7: Appendix G.

### Eligibility criteria

This review evaluates the effectiveness of survey methods, strategies, and incentives (hereafter “survey mode”) to improve adolescents’ response rates for surveys containing mental health, alcohol, and substance use questions. Adolescents were defined as those aged 12–19 years. It focuses on research conducted in a community setting published since 2007 (when smart phones were introduced). The outcome measures are:

- Survey response rates: the percentage of individuals who returned a completed survey, by survey mode;
- Mental health variation (i.e., self-reported prevalence) by survey mode. For example, depression scores or alcohol use rates reported for survey modes;
- Participant variations (e.g., gender differences) in self-reported mental health scores by survey mode.

Additional file 1: Appendix A present the review’s eligibility criteria and a glossary of definitions.

### Search strategy

One information specialist (EH) developed the search strategy, and a second peer reviewed it using the six domains of the PRESS guidelines [20]. Following a pilot search in the Cochrane Central Database of Controlled Clinical Trials (Wiley), an adapted search strategy was run in APA PsycINFO (Ovid), MEDLINE (Ovid) and Scopus (Elsevier) on May 13, 2022. Backwards and forwards citation searching were undertaken with last searches undertaken on June 28, 2022. Full searches are presented in Additional file 2: Appendix B.

### Study selection

**Fig. 1:**
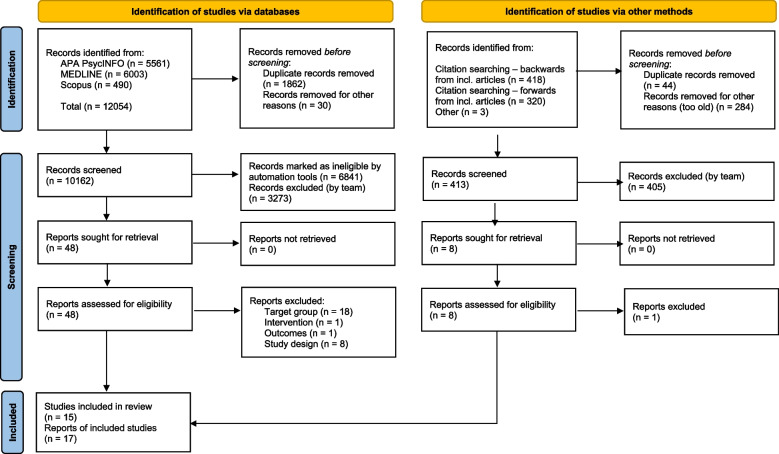
PRISMA diagram for the study identification and selection

We deduplicated records in EndNote and screened records in EPPI Reviewer 4 [21]. Two reviewers (JB, JFME) independently piloted the screening, using machine learning functions in EPPI-Reviewer combined with human assessment (see Additional file 2: Appendix B). Randomised controlled trials (RCTs) and non-randomised studies of interventions were screened first, and once we identified more than five (pre-specified) RCTs, screening for other study designs was stopped. The two reviewers screened titles and abstracts, and then each relevant full text, independently against the eligibility criteria. A third reviewer adjudicated disagreements. Figure 1 shows the search and screening, and Additional file 2: Appendix B lists the excluded studies.

For studies reported in several documents, all related documents were identified and grouped together to ensure participants were only counted once.

### Data extraction

The two reviewers conducted double independent data extraction into Excel forms. A third reviewer adjudicated disagreements. We piloted data extraction on five studies (see Additional file 3: Appendix C).

### Risk of bias (quality assessment)

The two reviewers assessed studies’ risk of bias (RoB) independently using Cochrane’s RoB 2.0 [22]. Any financial and non-financial conflicts of interest reported in the studies were collected as a separate bias category outside of RoB 2.0 (see Additional file 3: Appendix C).

### Data synthesis

The protocol provides full details of the planned data synthesis [18]. We present a summary here.

We grouped studies by the type of survey modes. When two or more studies reported the same outcome and survey modes were deemed sufficiently homogeneous, we checked that the data direction permitted pooling. Where necessary to make the values meaningful, we arithmetically reversed scales. We included studies in the meta-analyses regardless of their RoB rating.

To assess statistical heterogeneity, we first checked our data for mistakes and then used the Chi^2^ test (threshold *P* < 0.10) and the I^2^ statistic following Cochrane Handbook recommendations [23]. In cases of considerable statistical heterogeneity (I^2^ > 70%) we did not conduct meta-analysis. Where there was less heterogeneity (I^2^ <  = 70%), we performed random effects meta-analysis using Review Manager 5.4.1. We also assessed studies’ clinical and methodological heterogeneity (participants, survey processes, outcomes, and other study characteristics) to determine whether meta‐analysis was appropriate.

Where statistical pooling was not feasible, we followed the Synthesis Without Meta-analysis guideline to report the results narratively [24]. For dichotomous outcomes (e.g., response rates and adolescents’ self-reported alcohol use) we calculated odds ratios (ORs) and their 95% confidence intervals (CIs) to estimate between-mode differences. We used the default weighting technique (e.g., Mantel–Haenszel) for dichotomous outcomes in RevMan software. For continuous outcomes, we estimated the difference between survey modes using Mean Differences (MDs) or Standardized Mean Differences (SMDs) if the same outcome was measured with different questionnaires. The standard deviation was not modified [25]. We planned subgroup analyses and a GRADE assessment [18]. Amendments to the protocol are in Additional file 7: Appendix G.

## Results

### Search and screening results

Database searches retrieved 12,054 records. We removed 1,892 duplicates. EPPI-reviewer 4 marked 6,841 records as ineligible (see Additional file 2: Appendix B). The team screened the titles and abstracts of 3,321 records and the full text of 48 documents, identifying ten eligible documents. Citation searches on ten eligible documents retrieved a further 740 records, which yielded six eligible documents. We identified one further document from reference lists. In total, this review included 15 studies (17 documents). Additional file 2: Appendix B shows the excluded studies. We did not identify any studies in languages we could not translate.

Figure 1 shows the PRISMA diagram.

### Details of included studies

Table 1 provides details of the included studies and Additional file 3: Appendix C shows the data extraction tables. The age distribution of participants in the studies varied, but most were aged 14 to 16 years. A smaller proportion of participants were aged < 14 years or > 16 years. The sex distribution in studies were generally even and ranged from 32% [26] to 58% [27]. Studies were conducted in both rural and urban areas and included a range of national and racial/ethnic representation. Although most studies took place within school settings, four of them [26, 28–30] were conducted in non-school environments. All the studies involved community (i.e., non-clinical) samples, but we note that the Pejtersen’s study [26] focused on a group of vulnerable children and youth.

**Table 1:** Characteristics of the included studies

**Study ID** (Author, year) Country and study design	Setting and participants’ details	**Survey modes and** instrument	**Outcome measures**
**Web vs paper-and-pencil administration (*n* = 9 studies in 10 publications)**			
**Denniston 2010** **Eaton 2010** USA Cluster RCT [31, 32]	85 schools in 15 states 5786 students in 9th- or 10th-grade	Mode 1: paper-and-pencil administration** (**PAPI) (*n* = 1729) Mode 2: Web (*n* = 4057) 2.1 In-class Web without skips (students could skip a question if they did not want to answer it) – used school’s computer lab (*n* = 1735) 2.2. In-class Web with skips– used school’s stationary computer lab (*n* = 1763) 2.3. On your own Web – school’s computer lab (*n* = 559) Youth Risk Behavior Surveys (YRBS) relevant survey questions: (a) Sadness—felt sad or hopeless almost every day for 2 or more weeks in a row (b) Suicide attempt—attempted suicide during the last 12 months	• Response rates • Mental health variations by survey mode
**Hamann 2016** Switzerland RCT [33]	Students in 2 classes in a secondary school	Mode 1: PAPI (*n* = 28) Mode 2: Web (*n* = 28) German version of Children’s Depression Inventory (CDI) and the Spence Children’s Anxiety Scale (SCAS) In both tools, higher scores represent worse anxiety/depression	• Mental health variations by survey mode
**Lygidakis 2010** Italy RCT [14]	190 adolescents from 3 seniors’ high schools	Mode 1: PAPI (*n* = 97) Mode 2: Web (*n* = 93) Ad hoc questionnaire based on the European School Survey Project on Alcohol and Other Drugs questionnaire (2003) and the fifth Doxa national survey	• Mental health variations by survey mode
**Mauz 2018** Germany RCT [30]	Children and adolescents from 20 municipalities in 5 federal states	11,140 randomized / 1194 adolescents analysed (4662 including parents) Adolescents (11–17 years old) Mode 1: PAPI (*n* = 895) Mode 2: Web (*n* = 299) Survey design: Single mode design (*n* = 344 adolescents) Sequential mixed-mode design (*n* = 269 adolescents) (Web = 148, 55% of participants) Concurrent mixed-mode design (*n* = 290 adolescents) (Web = 43, 15% of participants) Preselect mixed-mode design (*n* = 292 adolescents) (Web = 102, 35% of participants) The German Health Interview and Examination Survey for Children and Adolescents (KiGGS) The Strengths and Difficulties Questionnaire (SDQ)	• Response rates • Mental health variations by survey mode
**Miech 2021** USA RCT [34]	41,866 students in 8th, 9th, and 12th grades from 397 middle or high schools in 48 states	Mode 1: PAPI (*n* = 20,039) Mode 2: Web (*n* = 21,475) Monitoring the Future (MTF) school-based national survey	• Response rates • Mental health variations by survey mode
**Raat 2007** The Netherlands RCT [35]	Adolescents (13–17 years old) from 55 classes at various educational levels in seven secondary schools in rural and urban areas	Mode 1: PAPI (*n* = 458) Mode 2: Web (*n* = 475) The Child Health Questionnaire Child Form (CHQ-CF): has 16 items (higher scores mean better mental health; e.g., children feel peaceful, happy, and calm all the time)	• Mental health variations by survey mode
**Raghupathy 2013** USA RCT [36]	Adolescents from a predominantly rural school district in Washington State	Mode 1: PAPI (*n* = 181) Mode 2: Web (*n* = 160) Youth Risk Behavior Surveys (YRBS) survey: 17 items on alcohol use and risk indicator measures including short and long-term use, binge drinking, and perception of harm	• Mental health variations by survey mode • Participant variations by survey mode
**Trapl 2007** USA RCT [37]	275 seventh grade students from Seven Cleveland Municipal School District (CMSD) K-8 schools	Mode 1: PAPI (*n* = 90) Mode 2: Web (*n* = 185) 2.1 Personal Digital Assistant (PDA) (*n* = 93) 2.2 Audio-enhanced personal digital assistant (APDA) (*n* = 92) Ad-hoc questionnaire, 178-question survey	• Response rate (assumed 100%) • Mental health variations by survey mode
**van de Looij-Jansen 2008** The Netherlands RCT [16]	551 students in third grade classes from 5 secondary schools	Mode 1: PAPI (*n* = 261) Mode 2: Web (*n* = 270) The Dutch self-report version of the Strengths and Difficulties Questionnaire (SDQ): sum score 0–10; higher scores mean worse mental health The Child Health Questionnaire (CHQ). Nine items about feelings and moods	• Response rates • Mental health variations by survey mode • Participant variations by survey mode
**Telephone interview vs postal questionnaires (*n* = 2)**			
**Erhart 2009** Germany RCT [29]	42 municipalities 1737 children and their parents	Mode 1: Telephone (*n* = 825) Mode 2: Postal/mail survey (*n* = 912) Strengths and Difficulties Questionnaire (SDQ): total score, higher scores indicate worse mental health, emotional symptoms, conduct problems, hyperactivity, peer relationship problems, and prosocial behaviour: scores 1 to 4 added together to generate a total difficulties score (based on 20 items)	• Response rates • Mental health variations by survey mode
**Wettergren 2011** Sweden RCT [28]	585 adolescents from three public healthcare regions (South, Uppsala / Orebro (Middle) and North)	Mode 1: Telephone (*n* = 300) Mode 2: Postal/mail survey, PAPI (*n* = 285) Short Form 36 (SF-36): mental health component scores, higher scores indicate better mental health The Hospital Anxiety and Depression Scale (HADS): a fourteen-item scale with seven items that relate to anxiety and seven that relate to depression; Lower scores indicate better mental health status	• Response rates • Mental health variations by survey mode • Population variations by survey mode
**Active vs Passive parental consent (*n* = 1)**			
**Courser 2009** USA RCT cluster [38]	14 school districts in Kentucky Students in grades 6th through 12^th^	Mode 1: Active consent (*n* = NR) Mode 2: Passive consent (*n* = NR) Kentucky Youth Outcomes (KYOS) Survey. Measure of risk and protective factors, behaviours related to alcohol, tobacco, and other drugs, and school safety issues	• Response rates • Mental health variations by survey mode • Population variations by mode
**Web first vs in person first interview (*n* = 1)**			
**McMorris 2009** USA RCT [27]	10 suburban public elementary schools in the Pacific Northwest school district 386 students enrolled in 1^st^ and 2^nd^ grade and followed up every spring	Mode 1: In-person first (*n* = 189) Mode 2: Web first (*n* = 197) Raising Healthy Children (RHC) Project: 14 items on substance use and sexual risk behaviour	• Response rates • Mental health variations by survey mode
**Voucher vs non-voucher (*n* = 1 reported in 2 documents)**			
**Pejtersen 2020** Denmark RCT [26, 39]	262 children and youth (vulnerable and lonely) from 10 municipalities	Postal questionnaire Mode 1: Voucher (*n* = 143)—supermarket voucher worth €15 Mode 2: Non-voucher (*n* = 119) Ad-hoc questionnaire on participants’ life situation: family and housing; education and training; sport and leisure time; relation to friends; drug use; and strengths and difficulties The Danish version of the Strengths and Difficulties Questionnaire (SDQ): emotional symptoms subscale (sum score 0–10; higher scores mean worse mental health)	• Response rates • Mental health variations by survey mode
**Internal supervision vs external supervision (*n* = 1)**			
**Walser 2012** Switzerland Cluster RCT [40]	80 classes in public high schools from the Canton of St. Gallen	Mode 1. External supervision (*n* = 40 classes, 598 students) Mode 2. Internal supervision (*n* = 40 classes, 599 students) Computer-assisted-self-interviewing (CASI) using a program from NETQ	• Mental health variations by survey mode

The fifteen studies investigated six comparisons:

1. Paper-and-pencil (PAPI) survey administration versus web administration (*n* = 9 in 10 documents)
2. Telephone interviews versus postal questionnaires (*n* = 2)
3. Active versus passive parental consent (*n* = 1)
4. Web first versus in-person first interviews (*n* = 1)
5. Vouchers versus no vouchers (*n* = 1 in 2 documents)
6. Internal supervision versus external supervision (*n* = 1)

### Risk of bias

Overall, study authors provided little information on their research methods resulting in several unclear domains that raised concerns about risk of bias. The main issues identified related to the randomisation process, measurement of the outcomes, and selective reporting of results. We classified three cluster RCTs [31, 32, 38, 40] and three parallel RCTs [26, 35, 37, 39] as high RoB. There were some concerns with nine [14, 16, 27–30, 33, 34, 36] parallel RCTs (see Additional file 4: Appendix D). RoB for each study is presented below.

### Outcomes

This section presents the study results and the meta-analyses. Additional file 6: Appendix F contains additional forest plots. We describe the results narratively without prioritization or hierarchy. We did not contact study authors for missing/additional data. Caution is advised when interpreting the meta-analyses because of studies’ quality/RoB and imprecision.

The considerable statistical heterogeneity (I^2^ > 70%) in the data for the two largest comparisons (1 and 2) precluded a meta-analysis of response rates. The studies showed divergent effect estimates, which may be explained by their different outcome measures. There were differences inherent to the study designs with cluster RCTs adjusted for clustering. There were important differences in the survey implementation procedures, including different interfaces, skipped questions, confidentiality measures and different degrees of supervision. Ignoring these considerations would have resulted in pooled analyses prone to misleading inferences.

#### Comparison 1: paper-and-pencil versus web-based administration mode

Nine studies (ten documents) compared PAPI surveys to web-based surveys [14, 16, 30–37]. The studies included one cluster RCT with high RoB, three RCTs with high RoB and five RCTs with RoB concerns.

##### *Response rate*

**Fig. 2:**
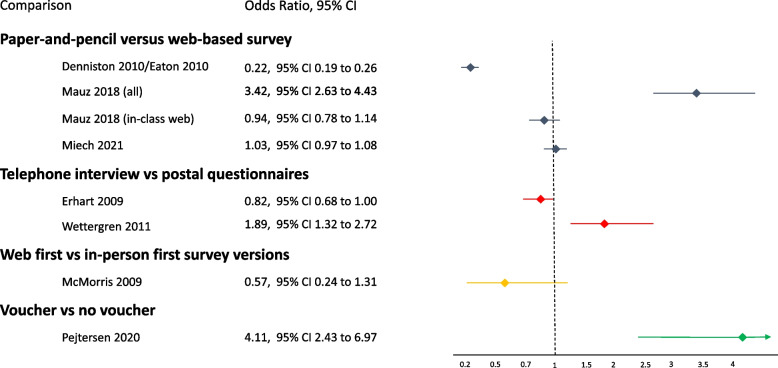
Odds ratios for various survey delivery mode comparisons: Adolescents’ response rates (results not pooled)

Five studies reported response rate [16, 30–32, 34, 37]. Three studies reported between-group differences [30–32, 34], but because of considerable heterogeneity (I^2^ > 90%) we present the effect estimates for each study separately (Fig. 2). Van de Looij-Jansen [16] reported a narrative summary rather than outcome data. Trapl [37] reported a 100% response rate.

Denniston [31], reported a cluster RCT in two documents [31, 32] and accounted for clustering in the analyses. Therefore, we did not conduct design effect adjustment [41]. The odds of response increased by nearly 80% for PAPI compared with a web mode (OR 0.22, 95% CI 0.19 to 0.26; *n* = 7747). Participants could skip questions in some of the modes (“with skip patterns”). Treated as an independent intervention arm, the group “on your own” web without skip patterns had the lowest response rate (28%; 559/1997) compared with the other web formats (in-class web without skips and with skips) and markedly lower odds of response relative to PAPI (OR 0.04, 95% CI 0.03 to 0.04). Low odds of response affect the pooled rates among the web survey modes. The pooled response rate for the two web in-class modes (with and without skips) was 90.7%, which was no different to the PAPI response rate (OR 0.94, 95% CI 0.78 to 1.14; *n* = 5750).

Mauz [30] explored three survey modes that we combined into an “overall web mode”. Each mode included varying proportions of participants receiving PAPI surveys or web surveys (see Table 1), but separate data for web participants were not reported. The odds of response decreased by nearly 70% when using PAPI compared with a web mode (OR 0.29, 95% CI 0.23 to 0.38; *n* = 1195) [30].

Miech [34] found evidence of no effect on response rates for PAPI compared with web mode (electronic tablets) (OR 1.03, 95% CI 0.97 to 1.08; *n* = 41,514).

Van de Looij-Jansen [16] reported an overall response rate of 90%, with no difference between PAPI or web modes (data not reported) and Trapl [37] reported 100% response rate.

##### Mental health variation by mode of survey delivery

Nine studies (ten documents) reported between-modes variations in point estimates for various mental health and substance use scores at the time of survey completion [14, 16, 30–37].

**Fig. 3:**
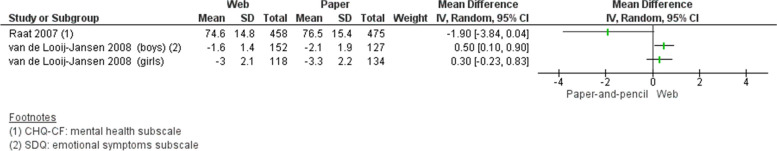
Mean differences for paper-and-pencil versus web administration survey delivery modes: Adolescents’ self-reported mental health

Two studies (considerable heterogeneity: I^2^ = 82%) of Dutch adolescents from secondary schools in rural and urban areas reported between-modes variations for adolescents’ mental health scores (Fig. 3) [16, 35]. Raat [35] reported that for the mental health subscale of the Child Health Questionnaire (CHQ-CF), PAPI mode participants had slightly lower scores compared with web users (MD -1.90, 95% CI -3.84 to 0.04; *n* = 933). Conversely, van de Looij-Jansen [16] reported no between-mode variations in self-reported total scores for the Strength and Difficulties Questionnaire (SDQ). Boys tended to report better mental health scores when completing surveys using PAPI than the web (MD 1.0, 95% CI -0.10 to 2.10; *n* = 279).

**Fig. 4:**
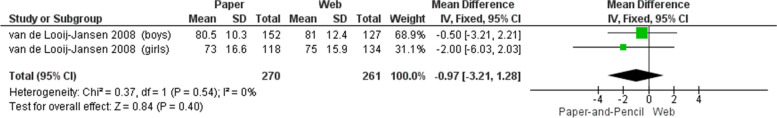
Mean differences for paper-and-pencil versus web administration survey delivery modes: Adolescents’ psychological wellbeing (nine items about feelings and moods derived from the CHQ-CF)

Two studies estimated between-mode variations for adolescents’ self-reported psychological wellbeing scores [16, 30]. Mauz [30] reported the number of adolescents experiencing favourable psychological wellbeing, expressed as *t* values, using the KIDSCREEN (the Health-Related Quality of Life Questionnaire for Children and Adolescents aged from 8 to 18 years, Questionnaires—kidscreen.org). The narrative findings indicated that psychological wellbeing was the same for both PAPI and web-based questionnaire modes (PAPI 50.5% vs web 49.3% (*n* = 1194), *P* = 0.07 adjusted with Bonferroni correction). Similarly, van de Looij-Jansen [16] reported no between-mode variations in mean scores of adolescents’ self-reported psychological wellbeing obtained from nine items about feelings and moods from the CHQ-CF (MD pooled for boys and girls -0.97, 95% CI -3.21 to 1.28; *n* = 531) (Fig. 4).

Denniston [31] found evidence of no between-mode estimate variations for adolescents’ self-reported sadness (OR 1.02, 95% CI 0.90 to 1.15; *n* = 5786) or suicide attempts (OR 1.01, 95% CI 0.83 to 1.24; *n* = 5786) measured using the Youth Risk Behavior Surveys [31, 32].

Hamann [33] found evidence of no between-mode estimate variations for adolescents’ self-reported anxiety (MD 1.65, 95% CI -5.18 to 8.48; *n* = 56) or depression (MD 0.78, 95% CI -1.54 to 3.10; *n* = 56) measured using the Spence Children’s Anxiety Scale (SCAS) and the German version of the Children’s Depression Inventory (CDI) [33].

**Fig. 5:**
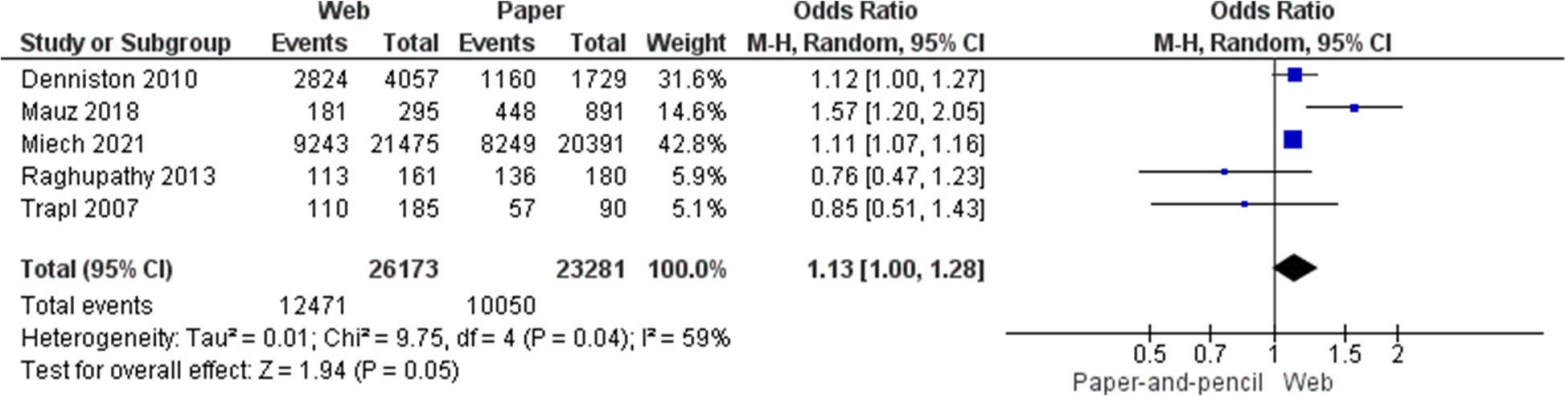
Odds ratios for paper-and-pencil versus web administration of surveys: Adolescents’ self-reported lifetime alcohol use

Six studies (7 documents) reported adolescents’ self-reported lifetime alcohol use [14, 30–32, 34, 36, 37]. Lygidakis [14] reported on adolescents who said they “*have been drunk*” and therefore we did not pool this study with studies of lifetime use. In Lygidakis [14], lifetime estimates of self-reported alcohol use were 11% lower in the PAPI group compared with the web survey group (OR 0.89, 95% CI 0.79 to 1.00; *n* = 190). A pooled analysis of five studies [30–32, 34, 36, 37] suggested that the odds of alcohol lifetime use were 13% higher among adolescents completing the web survey compared with those using PAPI (OR 1.13, 95% CI 1.00 to 1.28; *n* = 49,554); substantial heterogeneity was observed (I^2^ = 59%) (Fig. 5).

**Fig. 6:**
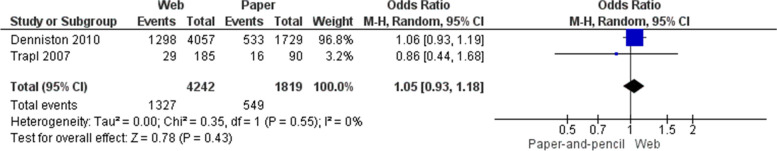
Pooled estimate variations for paper-and-pencil versus web administration of surveys: Adolescents’ self-reported lifetime marijuana use

A pooled analysis of two studies, Denniston [31] and Trapl [37], showed evidence of no between-mode estimate variations for adolescents’ self-reported marijuana use (OR 1.05, 95% CI 0.93 to 1.18; *n* = 6,061) (Fig. 6).

##### Participant variation by mode of survey delivery

Gender was the only participant characteristic for which the included studies reported disaggregated data. We calculated estimate variations by gender within studies rather than between survey mode comparisons.

**Fig. 7:**
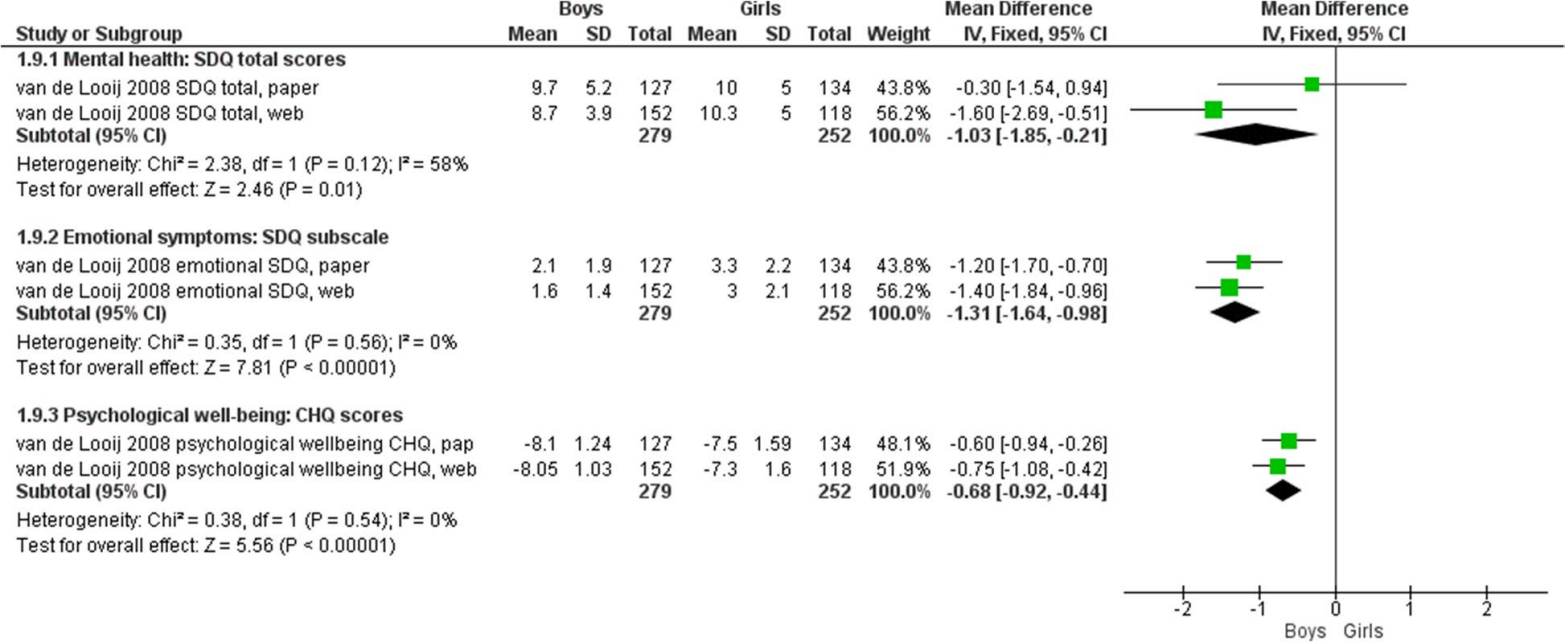
Mean difference by gender for paper-and-pencil and web administration of surveys: Adolescents’ self-reported mental health outcomes

In Van de Looij-Jansen [16], boys tended to report better mental health scores than girls for total mental health score, emotional symptoms, and psychological well-being. The largest and more precise difference was for emotional symptoms (pooled MD for both survey modes -1.31, 95% CI -1.64 to -0.98; *n* = 531), whereas the mental health total scores reported with the PAPI version of the SDQ proved to be the least precise (MD -0.30, 95% CI -1.54 to 0.94; *n* = 261). The absence of statistical heterogeneity in the results for emotional symptoms and psychological well-being suggests that boys reported better scores than girls regardless of the survey mode (Fig. 7).

**Fig. 8:**
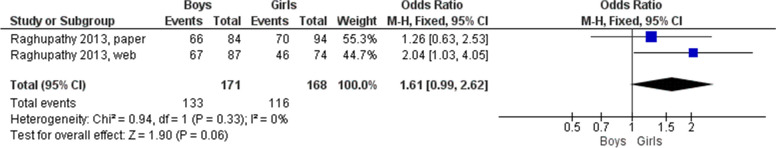
Odds ratios for gender variations for paper-and-pencil and web administration of surveys: Adolescents’ self-reported lifetime alcohol use

In Raghupathy [36], the odds of reporting lifetime alcohol use increased by more than one half in girls (OR 1.61, 95% CI 0.99 to 2.62; *n* = 339). Less precise estimate variations were observed when using PAPI vs web mode (Fig. 8).

#### Comparison 2: telephone interview vs postal questionnaires

Two studies reported outcome data for this comparison (*n* = 2322) [28, 29]. Trained interviewers performed the telephone interviews in both studies. Interviewers in Erhart [29] used computer-assisted telephone interviews whereas in Wettergren [28] interviewers were trained to read the questions aloud and record participants’ answers. There were concerns for RoB for both studies.

##### *Response rate*

We did not pool the response rates due to considerable heterogeneity (I^2^ > 90%); the studies are presented separately [28, 29]. The studies reported opposing results (Fig. 2). Erhart [29] reported a 41% completion rate for telephone interviews compared with 46% for postal questionnaires (OR 0.82, 95% CI 0.68 to 1.00; *n* = 1,737), whereas Wettergren [28] reported a response rate of 77% for telephone interviews and 64% for postal questionnaires (OR 1.89, 95% CI 1.32 to 2.72; *n* = 585).

##### *Mental health variation by mode of survey delivery*

**Fig. 9:**
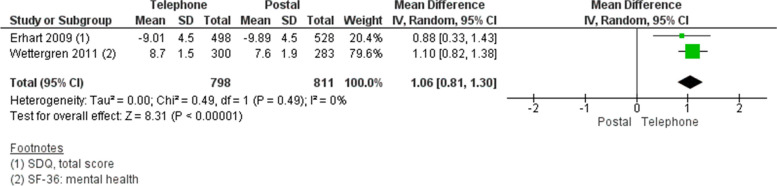
Pooled mean difference for survey delivery by telephone interview versus postal questionnaires: Adolescents’ self-reported mental health

The studies evaluated the effect of differences in survey mode on estimate variations of adolescents’ self-reported mental health measured by the SDQ total score [29] and the mental health component of the RAND 36-Item Short Form Health Survey (SF-36) measure [28]. We converted the data in Wettergren [28] to a zero to 10 scale to obtain a more homogenous pooled analysis. In the meta-analysis, adolescents reported 1.06 points better mental health when a telephone interview was used (MD 1.06, 95% CI 0.81 to 1.30; *n* = 1,609) (Fig. 9).

Wettergren [28] found evidence of no estimate variation for adolescents’ self-reported anxiety (MD -0.60, 95% CI -1.21 to 0.01; *n* = 580) and a small estimate variation for self-reported depression on the Hospital Anxiety and Depression Scale (HADS) favouring telephone interviews relative to postal questionnaires (MD -0.50, 95% CI -0.94 to -0.06; *n* = 585).

##### Participant variation by mode of survey delivery

**Fig. 10:**
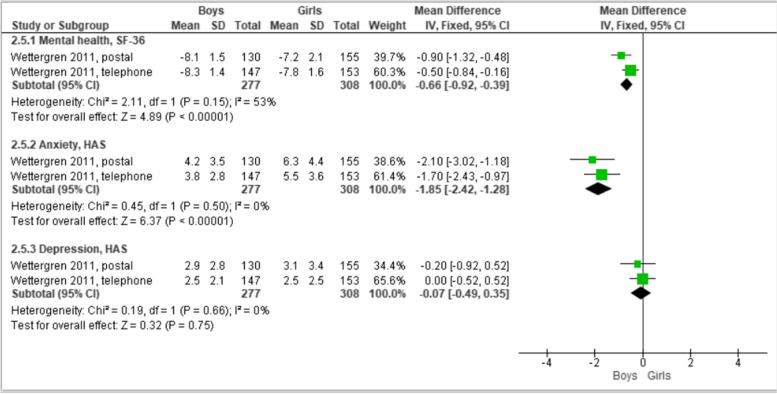
Pooled mean differences by gender for survey delivery by post and telephone: Adolescents’ self-reported mental health

Wettergren [28] reported participants’ gender differences in self-reported estimate variations of mental health (SF-36) alongside anxiety and depression (both measured with the HADS). Boys tended to report better mental health (SF-36) and anxiety (HADS) scores than girls, with the largest gender difference in anxiety (MD -1.85, 95% CI -2.42 to -1.28, *n* = 585) [28]. Postal questionnaires seem to result in a larger gender difference in self-reported mental health scores compared with telephone questionnaires (I^2^ = 53%). No differences between survey modes were observed for anxiety scores (I^2^ = 0%). Boys and girls reported similar depression scores (MD -0.07, 95% CI -0.49 to 0.35; I^2^ = 0%) for both survey modes (Fig. 10).

#### Comparison 3: active vs passive parental consent.

One cluster RCT compared schools randomised into groups where adolescents required active parental consent to undertake the survey or where passive parental consent was accepted [38]. The study had high RoB.

District schools assigned to passive parental consent achieved a response rate of 79% compared to 29% achieved by schools assigned to active consent mode (*p* = 0.001, number of participants per mode not reported) [38].

Courser [38] did not report any mental health variation or participant variations by survey mode.

#### Comparison 4: web first vs in-person first survey versions

One RCT [27] investigated the order of survey delivery. One group of students was offered an in-person survey, with web follow-up in case of non-response. A second group was asked to complete a web survey first, with in-person survey in case of non-response. There are some concerns over the study’s RoB.

McMorris [27] found evidence of no difference in response rates between adolescents completing a web survey first or an in-person survey first (OR 0.57, 95% CI 0.24 to 1.31; *n* = 386) (Fig. 2).

McMorris [27] found evidence of no difference on adolescents’ self-reported lifetime alcohol use (OR 0.84, 95% CI 0.55 to 1.27; *n* = 359) or lifetime marijuana use (OR 0.65, 95% CI 0.41 to 1.01; *n* = 359) between the two survey modes. McMorris [27] did not report on participant variations by survey mode.

#### Comparison 5: voucher vs no voucher

One RCT [26] (reported in two documents) investigated whether an unconditional monetary incentive (a supermarket voucher) increases the response rate among vulnerable children and youths receiving a postal questionnaire [26, 39]. The study was classified as high RoB.

Pejtersen [26] found that the monetary incentive yielded a response rate of 76% versus 43% without the incentive (OR 4.11, 95% CI 2.43 to 6.97; *n* = 262) (Fig. 2).

The study also found that offering a voucher made no difference to adolescents’ self-reported emotional symptoms compared with no voucher (MD -0.70, 95% CI -1.58 to 0.18; *n* = 156) measured using the emotional symptoms subscale of the SDQ [26, 39]. Pejtersen [26] did not report on participant variations by survey mode.

#### Comparison 6: internal versus external supervision

One Swiss cluster-RCT evaluated the effect of external supervision (by a senior student or researcher) compared to internal supervision (by the teacher) when students completed online interviews [40]. The study was classified as high RoB.

Walser [40] only reported outcomes relevant to mental health variations, finding evidence of no variations in adolescents’ self-reported lifetime alcohol use according to the survey mode (OR 1.08, 95% CI 0.79 to 1.47; *n* = 1,197).

### Subgroup and sensitivity analyses

There were too few studies, and no quasi-RCTs, to complete the planned subgroup and sensitivity analyses.

### Reporting bias assessment

We could not assess reporting biases, because too few studies were available (i.e., less than 10 studies) for each comparison [23].

### Certainty assessment

We opted not to perform a GRADE assessment due to the limited quantity of studies for each comparison under consideration and the mixed quality of studies.

## Discussion

This review identified fifteen RCTs that investigated six different comparisons among adolescents. Although the included studies were of mixed quality, several effective methods and strategies to improve adolescents’ response rates to mental health surveys were identified. Findings show that response rates varied with survey mode, consent type, and incentives.

Comparisons of web versus PAPI mode yielded discrepant findings that must be interpreted in relation to survey delivery context. One study showed that postal invitations to a web survey was associated with higher response rates compared to PAPI mode [30], possibly due to the additional effort required to return the completed PAPI survey by post. In contrast, there were no significant differences in response rates for web and PAPI modes conducted in classrooms during school hours [16, 31, 32, 34]. However, one study showed that inviting adolescents to complete a web survey on their own (at home within 2–3 weeks following the invitation) dramatically decreased response rates compared with completing PAPI or web surveys at school (28% vs. ~ 90%) [31, 32]. These findings show that response rates may vary according to both delivery mode and context. A previous meta-analysis showed that web surveys yield lower response rates (on average 12 percentage points) than other modes [12]. However, this review did not focus specifically on adolescents. More studies are needed to determine whether response rates among adolescents differ between web and PAPI surveys delivered outside school.

Conflicting evidence was found for telephone interview surveys compared to postal PAPI surveys. One study found significantly higher response rates (77% vs 64%) for telephone interview surveys [28], while another found significantly but marginally (48% vs. 43%) higher response rates for postal PAPI surveys [29]. The reasons for these opposing findings are unclear, but other contextual factors may play a role such as the age of the studies (conducted before 2010) reflecting potential time related differences in attitudes towards telephone interviews and postal PAPI surveys. One study [27] found that response rates did not differ significantly when comparing a web survey and follow-up in-person interview for non-responders with in-person interview and follow-up web survey for non-responders. Administering a web survey first is a cost-saving approach which is unlikely to adversely impact adolescents’ response rates.

One study showed that unconditional monetary incentives (i.e., voucher) increased response rates by 33 percentage points [26], supporting a prior review on postal surveys [42]. Interestingly, evidence favours monetary incentives *unconditional* on response compared with similar incentives *conditional* on response to improve response rates [11, 42]. In contrast, a recent meta-analysis [12] concluded that incentives had no effect on response rates in web surveys. These discrepant findings may indicate that incentives matter less for response rates in web surveys compared to other modes. Our review also identified one study showing that passive parental consent achieved more than double the response rate of active consent (79% vs. 29%) [38]. A prior meta-analysis of studies found similar evidence in favour of passive parental consent [43]. If ethical and data protection considerations permit, using passive parental consent may boost response rates substantially.

Survey mode influenced mental health scores in certain comparisons. We found no evidence of effect on self-reported mental health scores (across a range of measures) between PAPI and web surveys [16, 30–32, 34–37]. However, our pooled analysis of lifetime alcohol use showed 13% higher use when a web mode was used compared to a PAPI mode. This could possibly be attributed to differential response rates, for example if heavy drinkers are less likely to respond to a PAPI compared to web survey. In contrast, two studies indicated that lifetime marijuana use did not differ between web and PAPI survey modes [31, 32, 37]. The reasons for such differences are unclear and should be further researched. Telephone interview compared with postal PAPI surveys was associated with slightly better mental health scores [28, 29]. These differences were quite small and probably of limited practical significance [28]. Nonetheless, survey designers should be aware that adolescents may report fewer mental health problems in telephone interviews. Such findings may be due to differential response rates as already mentioned, for example if those with mental health problems are less likely to respond to telephone surveys compared to PAPI surveys. Another reason may be that adolescents are less willing to report such problems directly to another person. The added anonymity of non-telephone surveys may encourage adolescents to provide more genuine responses to sensitive questions concerning their mental health. A study that compared supervision by either teachers or researchers during an in-class web survey [40] found no significant differences in mental health scores, which suggests that the choice of supervision personnel does not impact responses.

There was little evidence of differences between gender and survey characteristics on mental health scores. While several studies highlighted that males report better mental health than females [16, 28], there was no indication that specific survey modes impacted males’ and females’ mental health differentially (i.e., no interaction effect). Many studies did not report mental health scores separately for males and females.

Our review complements earlier reviews of factors that influence response rates [11, 12, 42–44]. Together, these reviews provide useful information regarding how to design surveys to maximise response rates. The extent to which their findings are generalizable to adolescents in recent decades is unclear. Our own review show that relatively few studies have focused specifically on adolescents. Nevertheless, many of our findings are in line with those outlined in previous reviews. One outstanding question is whether web surveys yield lower response rates than other modes also for adolescents. The studies included in our review highlights the need to consider contextual factors when comparing response rates between surveys. For example, survey mode may have less impact on response rates in class-room settings. Our findings highlight the need for more studies to provide high-quality evidence of methods and strategies to ensure adequate response rates in mental health surveys of adolescents. This is particularly important given the present worldwide focus on adolescent mental health and the decreasing response rates in surveys.

Although we found relevant RCTs, they were of insufficient quality to draw firm conclusions. The studies in some comparisons showed considerable heterogeneity and meta-analysis was not feasible for most comparisons. For several comparisons, only one or two studies were available. In RCTs where one survey mode was superior to another, the results need to be confirmed with better conducted (and/or reported) studies.

The studies had a range of differences that reduce the comparability of studies and the generalisability and strength of our findings. Various questionnaires were used, differing greatly in content, length, and appearance. Questionnaires were managed in different ways, for example some used skips to ensure confidentiality, and some did not permit the questions to be read aloud during interview. Different methods were used to deliver questionnaires: postal, in the classroom, or sent to parents. The studies investigated a mix of outcomes using a range of tools and with study-specific adaptations in some cases.

The median publication year of the studies is 2010. The inclusion of older RCTs may mean that in a world of high internet and smart phone usage, the applicability of the earlier findings may be weakened.

Key strengths of this review include the team’s expertise in synthesis methods, topic area, information retrieval, and machine learning. We identified a substantial number of RCTs in adolescent populations, some with many participants, using an extensive search in databases augmented by forwards and backwards citation searching.

Although it is not usually common practice to search for outcomes in literature searches for reviews of effect of interventions [45], given the challenges of searching for this review topic, we considered it necessary to reduce the screening burden by including the concept of outcomes in our search. This approach may have lowered the search sensitivity where authors did not mention outcomes of interest in the abstract [46] and may also have introduced publication bias, because outcomes with positive results might be more likely to reported in the abstract than negative results [47]. Our citation searches should have mitigated both issues somewhat since they rely on publications citing each other, rather than containing specific words.

The review used machine learning for study selection reducing the study selection workload by 95%. Our experience confirms the widely documented potential of automated and semi-automated methods to improve systematic review efficiency [48, 49]. The workload savings enabled us to spend more time in discussions with content experts.

The review results are affected by statistical heterogeneity in the analyses, which may be due to methodological and clinical heterogeneity in the variables, as well as the large variability in the design and conduct of the studies. There were not enough studies to explore heterogeneity using subgroup and sensitivity analyses, nor to test for publication bias. In many instances, results come from a single study, which greatly reduces the applicability of the findings considering none of the studies had low RoB.

We limited eligible studies to those undertaken in high income countries and as a result we cannot generalize our findings to low- or middle-income countries. The body of evidence comes from nationwide surveys in schools in the USA and Europe.

### Implications for research

There is a need for more evidence on how best to identify records which report research into modes of data collection.

Some of the analyses showed unexpected results which might merit further research. These include lifetime alcohol use being higher when a web survey was used compared to PAPI, although there was no difference for lifetime marijuana use. Also, the evidence of differences in reported mental health for telephone compared with web surveys merit further investigation. Whether and in what situations web surveys yield poorer response rates compared to other modes in adolescents should also be investigated in future studies.

The absence of research evidence on the impact of survey mode on mental health scores by gender or other demographic characteristics, suggests this area could merit research.

There is a need for research that could better reflect the current situation where adolescents’ use of the internet and smart phones is widespread.

### Implications for practice

Survey designers must balance practical concerns against the sampling, non-response and measurement error associated with specific design features. This review, and others, highlight methods and strategies that may improve survey response rates among adolescents with minimal impact on the assessment of mental health status [11, 12, 42]. Based on the poor reporting in the included studies, authors should be encouraged to register their trials and make their protocols publicly available. Authors and journal editors should follow the CONSORT reporting guidelines [50].

## Conclusions

Despite the absence of low RoB studies, few studies for some comparisons and the focus on research undertaken in high income countries, there are methods and strategies that could be considered for improving survey response rates among adolescents being surveyed about mental health and substance use. For example, the use of monetary incentives may lead to higher response rates. Our review show that survey mode has limited impact on response rates in surveys delivered in school settings. Outside school settings, web surveys may be superior to other modes, but more research is needed to determine this. More studies using controlled designs are needed to further identify effective methods and strategies to ensure adequate response rates among adolescents. Some studies indicate that mental health scores may differ between certain survey modes. Finally, there was limited evidence on any differences between gender and survey characteristics on mental health scores.

### Supplementary Information

**Additional file 1. **Eligibility criteria and Glossary.

**Additional file 2. **Search strategies and lists of excluded studies.

**Additional file 3. **Detailed data extraction for the included studies.

**Additional file 4. **Risk of bias assessment.

**Additional file 5. **PRISMA checklist.

**Additional file 6. **Additional Forest plots.

**Additional file 7. **Protocol changes.

## Data Availability

The templates for data collection, the extracted data and the data used for all of the analyses are available from the main author upon reasonable request.

## Abbreviations

PRISMA: Preferred Reporting Items for Systematic Reviews and Meta-Analyses
RCT: Randomised controlled trial
RoB: Risk of bias
OR: Odds ratio
SMD: Standardized mean difference
GRADE: Grading of Recommendations, Assessment, Development, and Evaluations
MD: Mean difference
PAPI: Paper-and-pencil
CHQ-SF: Child Health Questionnaire
CDI: Children’s Depression Inventory
SCAS: Spence Children’s Anxiety Scale
SF-36: RAND 36-Item Short Form Health Survey
HADS: Hospital Anxiety and Depression Scale
