# Supplementary material for: Methods, strategies, and incentives to increase response to mental health surveys among adolescents: a systematic review
Source: BMC Med Res Methodol. 2023 Nov 16;23:270. doi: 10.1186/s12874-023-02096-z (PMC10652438; doi:10.1186/s12874-023-02096-z)
Supplement: Supplementary file 1 — Additional file 1. Eligibility criteria and Glossary. [file 12874_2023_2096_MOESM1_ESM.docx]

**Appendix A Details of the systematic review methods and glossary of terms**

The detailed review methods are available in the protocol (ref #10828), but the review eligibility criteria are presented here.

**Table A.1: Eligibility criteria for the review of methods, strategies and incentives to improve adolescents’ participation in surveys containing mental health and substance use questions**

| **Criterion** | **Eligible studies** | **Ineligible studies** |
| --- | --- | --- |
| **Type of studies** | - Completed randomized-controlled trials (RCTs) or quasi randomized trials (i.e., that is have used alternation, date of birth or case record number as a method of randomly allocating participants). If there were insufficient RCTs (fewer than 5) we planned to include non-randomized studies.      - Parental consent for participation may be mandatory in some countries. We planned to include studies where parents or guardians complete the questionnaire on behalf of or with the adolescent. | - Cohort studies with embedded randomised trials. - Editorials and comments were excluded at the search stage |
| **Type of data** | - Data that assessed the effects of methods, strategies, or incentives to increase adolescents’ response to surveys. - Studies addressing mental health including substance use (including all disciplines and disease areas). - Data for adolescents aged 12-19 years of all genders, ethnic and cultural groups. - Strategies used within real settings. - Conference abstracts were only considered if they provided additional information for trials already published in full. | - Studies of hypothetical survey studies (that ask potential adolescents whether they would take part in a survey if it was run but the survey does not actually exist). - Studies of retention strategies. - Studies in which ≥ 51% of participants are aged <10 or >25 years. |
| **Type of methods and comparisons** | - Any methods, strategies, or incentives designed to increase response rate, including - how to administer the study (e.g., format or appearance), content, and properties) - the context - communication (I.e., what are the participants told) - The methods can be compared to each other or to usual study procedures.   Any strategies and incentives identified during the review process will be included, but potential methods, strategies and incentives could include:   - **Motivational,** e.g. monetary incentives, gifts and non-monetary incentives - **Communication, e.g.** - Personalization of letters, birthday cards - Assurance of anonymity - Video conferencing (e.g., Zoom, Teams, Skype) - **Methodological, e.g.** - Alternative lengths or styles of questionnaire - Alternative format/delivery (paper-based, web based) - Alternative recruiting format/approaching participants (handed invitations, send out invitations by mail or email) - **Social, e.g.** - Scheduling research assessments in the company of other adolescents - Strategies encouraging family support. |  |
| **Outcomes** | - Response rate: including, but not limited to, proportion of completed survey/questionnaires returned after first round or all rounds if available - Mental health variation (I.e. prevalence) by mode of survey delivery. For example, depression scores or alcohol use rates identified for each of the different modes of survey completion - Participant variations (e.g. gender interactions) in self-reported mental health by mode of survey delivery. Including, but not limited to, features of the sample such as racial identity, geographical distribution, socio-economic characteristics, mental health status. This outcome did not consider mode of survey delivery. Where possible thorough descriptions were collected of survey/questionnaire design and strategies on recruitment. |  |
| **Setting** | - Studies assessing general mental health and/or substance use among an adolescent population within the community (e.g., high school, home). - Studies conducted among adolescents in high income countries according to the World Bank classification. | - Studies conducted primarily in institutional contexts, including healthcare facilities and criminal justice settings. - Studies conducted among adolescents in low- and middle-income countries |
| **Languages** | Spanish, Italian, French, Portuguese, Scandinavian languages, English  Articles meeting the criteria that we could not translate would be listed in the report |  |
| **Year** | 2007 (inception of the smart phone) to present | Studies published before 2007 |

## Glossary of terms

**Adolescent**: The WHO has traditionally defined adolescence to be the age between 10 and 19 years, youth between 15 and 24 years, and young people between 10 and 24 years (1). Adolescence can be further divided into early (10-13 years), middle (14-16 years), and late adolescence (17-19 years) according to Sawyer et al. (2)

**Bias**: A systematic error or deviation in results or inferences from the underlying ‘truth’. Biases include selection bias, performance bias, attrition bias, detection bias and reporting bias.

**Child**: Defined by the Convention on the Rights of the Child (1989) as a person younger than 18 years, unless majority (i.e., the legal threshold of adulthood) is attained at a younger age in a particular country (3).

**Cross sectional study**: A study that examines the relationship between diseases (or other health related characteristics) and other variables of interest as they exist in a defined population at a particular time.

**Estimate of effect**: The observed relationship between an intervention and an outcome expressed as, for example odds ratio, risk difference, risk ratio, hazard ratio, standardised mean difference, weighted mean difference, number needed to treat.

**Funnel Plot**: A graphical display of study precision such as the standard error plotted against effect size that can be used to investigate biases associated with small trials (including publication bias).

**Heterogeneity**: In systematic reviews heterogeneity refers to variability or differences between studies. A distinction is sometimes made between statistical heterogeneity (differences in the effect estimates), methodological heterogeneity (differences in study design) and clinical heterogeneity (differences in participants, interventions or outcome measures).

**Meta analysis**: Statistical techniques used to combine the results of two or more studies and obtain a combined estimate of effect.

**Prevalence**: the proportion of a population that has a specific characteristic in a given time period (4). Ways to estimate prevalence include:

- Point prevalence: the proportion of a population that has the characteristic at a specific point in time.
- Period prevalence: the proportion of a population that has the characteristic at any point during a given time period of interest. “Past 12 months” is a commonly used period.
- Lifetime prevalence: the proportion of a population who, at some point in life, has ever had the characteristic.

**Quasi randomized study**: unlike a true experiment, a quasi-experiment does not rely on random assignment. Instead, subjects are assigned to groups based on non-random criteria such as alternation, date of birth or case record number.

**Randomization:** The process of allocating participants to one of the groups of a randomized controlled trial using (i) a means of generating a random sequence and (ii) a means of concealing the sequence, such that those entering participants to a trial are unaware of which intervention a participant will receive. This should ensure that intervention groups are balanced for both known and unknown factors.

**Randomized controlled study:** These are trials where participants (or clusters) are randomly allocated to receive either intervention or control. If well implemented, randomization should ensure that intervention and control groups only differ in their exposure to treatment (5).

**Response rate**: The number of respondents who complete a questionnaire compared to the number assigned, usually expressed as a percentage (6).

**Survey:** An epidemiologic survey consists of simultaneous assessment of the health outcome and exposures as well as potential confounders and effect modifiers. A survey is considered a cross-sectional study. Some epidemiologists may call it a prevalence study (7).

**Sensitivity Analysis**: An analysis used to test the robustness of findings and determine how sensitive results are to the data that were included and/or the way that analyses were conducted.

**Teenager:** Refers to people aged 13–19 years. The term was first used in the USA in the 1920s and became widely used within popular culture after World War II.

**Young people:** A less formally defined term that generally refers to people aged 10–24 years, as does the composite term adolescents and young adults (8). When data are reported, the 10–24 year age range is increasingly being divided into three categories: 10–14 years (early adolescence), 15–19 years (late adolescence) and 20–24 years (young adulthood), to appropriately examine the extent of changes in health that take place during these years (9).

**Youth:** The United Nations defines youth as people aged between 15 years and 24 years, a definition made in the lead up to the International Youth Year of 1985.

**References**

**1.** World Health Organization. Health for the World’s Adolescents. A second chance in the second decade. Geneva, Switzerland: WHO; 2014.

2. Sawyer SM, Afifi RA, Bearinger LH, Blakemore S-J, Dick B, Ezeh AC, et al. Adolescence: a foundation for future health. The Lancet. 2012;379(9826):1630-40.

3. United Nations, United Nations Human Rights Office of the High Commissioner. Convention on the Rights of the Child. The Office of the High Commissioner for Human Rights; 2022. Available from: https://www.ohchr.org/en/instruments-mechanisms/instruments/convention-rights-child

4. National Institute of Mental Health. What is prevalence? Bethesda MD: National Institute of Mental Health. Available from: https://www.nimh.nih.gov/health/statistics/what-is-prevalence

5. National Insitute for Health and Care Excellence. Appendix D. Glossary of Study Designs. IN: Methods for the development of NICE public health guidance 3^rd^ ed. London: National Insitute for Health and Care Excellence; 2012 Available from: https://www.nice.org.uk/process/pmg4/chapter/appendix-d-glossary-of-study-designs#randomised-controlled-trial-rct

6. Organisation for Economic Co-operation and Development. Glossary of Statistical Terms. Response rate. Paris: Organisation for Economic Co-operation and Development;2005. Available from: https://stats.oecd.org/glossary/detail.asp?ID=7080

7. Penn State Eberly College of Science. Epidemiological Research Methods; Survey Study Design. State College: The Pennsylvania State University; 2022. Available from: https://online.stat.psu.edu/stat507/lesson/5/5.1

8. World Health Organization. The Second decade: improving adolescent health and development. Geneva: Department of Child and Adolescent Health and Development; 2001. Available from: https://apps.who.int/iris/handle/10665/64320

9. World Health Organization. Global health risks: mortality and burden of disease attributable to selected major risks. Geneva: World Health Organization; 2009. Available from: https://apps.who.int/iris/handle/10665/44203
