# Supplementary material for: Methods, strategies, and incentives to increase response to mental health surveys among adolescents: a systematic review
Source: BMC Med Res Methodol. 2023 Nov 16;23:270. doi: 10.1186/s12874-023-02096-z (PMC10652438; doi:10.1186/s12874-023-02096-z)
Supplement: Supplementary file 2 — Additional file 2. Search strategies and lists of excluded studies. [file 12874_2023_2096_MOESM2_ESM.docx]

**Appendix B: Search strategies, record screening and lists of excluded studies**

**B.1 Search strategies**

The information retrieval was conducted in several steps:

- A pilot search in the Cochrane Central Register of Controlled Trials (Wiley) was conducted on 6 May 2022 and the results were screened and discussed with the team
- Relevant articles from the pilot search were used to inform the development of the final search strategy
- A second information specialist peer reviewed the strategies prior to executing the main database searches in APA PsycINFO (Ovid), MEDLINE (Ovid) and Scopus (Elsevier) in May 2022.
- Two reviewers (JB, JFME, EH) screened title/abstract as well as full text.
- Following the selection process of articles from the main search, we used Web of Science (Clarivate) and Scopus (Elsevier) to collect references from included articles (backwards citations), and to screen title/abstract as well as full text for inclusion. The citation search was run June 20 2022. The citation search identified four new articles that met our inclusion criteria.
- We also consulted with the content expert and decided to run another iteration of the citation search, backwards (references in the included articles) for the four new records and forwards to identify articles citing the included articles. We used the SpiderCite tool ((Bond University, Institute for Evidence-Based Healthcare) for the second round of citation searching, which was conducted on June 28 2022

Although it is not usually common practice to search for outcomes in literature searches for reviews of effect of interventions (1), since this was a review of methods, we considered it necessary to limit our search by including the concept of outcomes. This option was selected to reduce the screening burden from articles where the topic of interest is not the survey method *per se*, as in this review, but studies using surveys or questionnaires to collect data on mental health issues. This approach may possibly lower the sensitivity of the search, and may also introduce publication bias, because outcomes with positive results might be more likely to reported in the abstract and thus captured in the retrieved records, than negative results (ref #10777; ref #10778). We believe that the variety of searches we have used should mitigate both the recall and bias issues somewhat.

The search strategies are shown below.

**Table B.1:** Pilot search in Cochrane Central Database of Controlled Trials (CENTRAL)

| **Cochrane Library**  Advanced search – Search manager  Date: 2022-05-06 | | |
| --- | --- | --- |
| #1 | [mh Adolescent] OR (adolescen* OR teen? OR teenager? OR youth OR "yong people"):ti,ab,kw OR (freshm?n OR high NEXT school* OR (second* NEAR/2 school*) OR sixth NEXT form OR sophomore?):ti,ab,kw | 152424 |
| #2 | (questionnaire? or survey*):ti,ab,kw or ("Child Behaviour Checklist" or "Hopkins Symptom checklist" or "Hospital anxiety and depression scale" or "Mood and feelings questionnaire" or "Strength and difficulties questionnaire"):ti,ab,kw | 180885 |
| #3 | ((interview*):ti,ab,kw or ("Child and Adolescent Psychiatric Assessment" or "Composite International Diagnostic Interview" or CIDI or "Development and Well-Being Assessment" or DAWBA or "Diagnostic Interview Schedule for Children" or "Preschool Age Psychiatric Assessment" or "Schedule for Affective Disorders and Schizophrenia" or "Kiddie-SADS" or "K-SADS" or "Structured Clinical Interview"):ti,ab,kw) NOT "motivational interviewing":ti | 40934 |
| #4 | [mh Methodology] OR [mh "Patient Selection"] OR [mh "Selection Bias"] OR (instrumentation OR non-respon* OR nonrespon* OR refusal* OR representativ* OR (response NEXT (bias OR frequenc* OR rate*)) OR return* OR sampl* OR (selection NEXT (bias OR error*))):ti,kw | 64604 |
| #5 | ((questionnaire* OR survey*) NEAR/10 (instrumentation OR non-respon* OR nonrespon* OR refusal* OR representative* OR response NEXT bias OR response NEXT frequenc* OR response NEXT rate* OR return* OR sampl* OR selection NEXT bias OR selection NEXT error*)):ti,ab | 7598 |
| #6 | (interview* NEAR/10 (instrumentation OR non-respon* OR nonrespon* OR refusal* OR representative* OR (response NEXT bias) OR (response NEXT frequenc*) OR (response NEXT rate*) OR return* OR sampl* OR (selection NEXT bias) OR (selection NEXT error*))):ti,ab | 1906 |
| #7 | [mh "Mental Health"] OR [mh "Adolescent Health"] OR (addict* OR ADHD OR adolescent NEXT health OR affective OR alcohol OR anxiety OR behavior* OR behaviour* OR cannabis OR cognitive* OR ((conduct OR disruptive OR oppositional NEXT defiant) NEXT disorder?) OR depress* OR distress* OR emotional* OR happiness OR hyperactivity OR maladjust* OR mari?uana OR mental* OR neuro-development* OR neurodevelopment* OR psychiatric* OR psychological* OR psychosis OR psychosocial* OR psychotic OR recilien* OR restless* OR self-esteem OR (self NEXT (harm OR injur*)) OR smoking OR social* OR substance OR suicid* OR tobacco OR wellbeing OR well-being):ti | 154627 |
| #8 | #1 AND (((#2 OR #3) AND #4) OR #5 OR #6) AND #7 with Publication Year from 2007 to present, in Trials | 553 |

PsycINFO

This strategy did not include a concept for mental health or substance use issues since the database is focused on psychological and psychiatric topics.

**Table B.2: PsycINFO search strategy**

| **APA PsycInfo <1806 to May Week 2 2022>**  Date: 2022-05-13 Ovid interface - advanced search | | |
| --- | --- | --- |
| 1 | "200".ag. or (adolescen* or teen? or teenager? or young people or youngster? or youth).ti,bt,ab,id. or (freshm?n or ((grammar or high or intermediate or middle or second*) adj2 school*) or gymnasia or gymnasium or highschool* or sixth form or sophomore?).ti,bt,ab,id. [age group; incl. schools] | 669216 |
| 2 | (exp Interviews/ or Questionnaires/ or Surveys/ or Mail Surveys/ or Online Surveys/ or Telephone Surveys/) and (Methodology/ or Patient Selection/ or Response Bias/ or Response Frequency/ or exp "Sampling (Experimental)"/) | 2825 |
| 3 | ((interview* or questionnaire? or survey?) adj10 (bias* or instrumentation or mode or modes or non-participat* or nonparticipat* or nonrepresentativ* or non-respon* or nonrespon* or over-represent* or overrepresent* or refusal* or representativ* or response frequenc* or response rate* or sampl* error* or sampling or selection error* or under-represent* or underrepresent*)).ti,bt,ab,id. | 32180 |
| 4 | 1 and (2 or 3) | 7078 |
| 5 | limit 4 to yr="2007 -Current" | 5561 |

MEDLINE

**Table B.3: MEDLINE search strategy**

| **Ovid MEDLINE(R) ALL <1946 to May 11, 2022>**  Date: 2022-05-13 Ovid interface - Advanced search | | |
| --- | --- | --- |
| 1 | Adolescent/ or (adolescen* or teen? or teenager? or young people or youngster? or youth).ti,bt,ab,kf. or (freshm?n or ((grammar or high or intermediate or middle or second*) adj2 school*) or gymnasia or gymnasium or highschool* or sixth form or sophomore?).ti,bt,ab,kf. [age group; incl. schools] | 2303297 |
| 2 | "Mental Health"/ or "Adolescent Health"/ or ((emotional* or mental* or psycho* or social*) adj3 (health? or well-being or wellbeing)).ti,bt,ab,kf. or (addict* or ADHD or adolescent health or affective or alcohol or anxiety or behavior* or behaviour* or cannabis or cognitive* or ((conduct or disruptive or eating or oppositional defiant) adj disorder?) or depress* or distress* or emotional* or feeling? or happiness or hyperactivity or maladjust* or mari?uana or mental* or mood or neuro-development* or neurodevelopment* or psychiatric* or psychological* or psychosocial* or recilien* or restless* or self-efficacy or self-esteem or (self adj (harm or injur*)) or smoking or social* or "strength and difficulties" or substance or suicid* or tobacco or wellbeing or well-being).ti,bt. | 1608622 |
| 3 | Cross-Sectional Studies/mt or Health Surveys/mt or "Surveys and Questionnaires"/mt or Interviews as Topic/mt or Interview, Psychological/mt or ((Cross-Sectional Studies/ or Health Surveys/ or "Surveys and Questionnaires"/ or Interviews as Topic/ or Interview, Psychological/) and (Bias/ or Methodology/ or "Patient Selection"/ or "Selection Bias"/)) | 15304 |
| 4 | ((interview* or questionnaire? or survey?) adj10 (bias* or instrumentation or mode or modes or non-participat* or nonparticipat* or nonrepresentativ* or non-respon* or nonrespon* or over-represent* or overrepresent* or refusal* or representativ* or response frequenc* or response rate* or sampl* error* or sampling or selection error* or under-represent* or underrepresent*)).ti,bt,ab,kf. | 74679 |
| 5 | 1 and 2 and (3 or 4) | 7676 |
| 6 | limit 5 to yr="2007 -Current" | 6003 |

SCOPUS

**Table B.4: SCOPUS search strategy**

| Scopus (Elsevier)  Date: 2022-05-13 Advanced search | | |
| --- | --- | --- |
| #1 | (TITLE-ABS-KEY(adolescen* OR teen? OR teenager? OR youngster? OR "young people" OR youth) OR TITLE-ABS-KEY(freshm?n OR ((grammar OR high OR intermediate OR middle OR second*) W/1 school*) OR gymnasia OR gymnasium OR highschool* OR "sixth form" OR sophomore?)) AND NOT INDEX(medline) | 625257 |
| #2 | (TITLE-ABS-KEY((emotional* OR mental* OR psycho* OR social*) W/2 (health? OR "well-being" OR wellbeing)) OR TITLE(addict* or ADHD or "adolescent health" or affective or alcohol or anxiety or behavior* or behaviour* or cannabis or cognitive* or ((conduct or disruptive or eating or "oppositional defiant") W/0 disorder?) or depress* or distress* or emotional* or feeling? or happiness or hyperactivity or maladjust* or mari?uana or mental* or mood or "neuro-development*" or neurodevelopment* or psychiatric* or psychological* or psychosocial* or recilien* or restless* or "self-efficacy" or "self-esteem" or (self W/0 (harm or injur*)) or smoking or social* or "strength and difficulties" or substance or suicid* or tobacco or wellbeing or well-being)) AND NOT INDEX(medline) | 1957750 |
| #3 | TITLE-ABS-KEY((interview* OR questionnaire? OR survey?) W/9 (bias* OR instrumentation OR mode OR modes OR nonparticipat* OR "non-participat*" OR nonrepresentativ* OR "non-respon*" OR nonrespon* OR "over-represent*" OR overrepresent* OR refusal* OR representativ* OR "response frequenc*" OR "response rate*" OR "sampl* error*" OR sampling OR "selection error*" OR "under-represent*" OR underrepresent*)) AND NOT INDEX(medline) | 31864 |
| #4 | (#1 AND #2 AND #3) | 572 |
|  | Filter on year of publication 2007-> | 490 |

**Citation searches**

Backwards citation (see above) on 12 eligible papers identified from the database searches retrieved a further 388 records. After removing duplicate records or those that were too old, 112 records progressed to screening.

In the second round of citation searching (see above), backwards citation searching on 4 papers retrieved 30 records and forwards screening of 16 papers identified 314 records. Following deduplication and the removal of older records, 298 records were screened.

**Reference checking**

When checking the included studies, we identified a clinical trial registry entry for one trial (2).

**B.2 Deduplication**

A RIS-file with included references is available on request from the authors.

We loaded search results from the bibliographic databases into EndNote (3) and conducted stepwise semi-automated deduplication using information in the author, title and DOI fields. Following deduplication, we loaded the unique references into EPPI-Reviewer (4).

The screening was piloted in 547 studies by two reviewers (JB and JFME) independently, using machine learning functions available in EPPI-Reviewer combined with human assessment.

**B.3 Record processing with EPPI-Reviewer**

First, we used the *Cochrane RCT classifier* (5) to automatically exclude studies classified with a 0-9% (low) probability of being RCTs. Second, the studies classified to be an RCT with more than 9% probability were screened with the *Priority screening* functionality. This semi-automated function uses machine-learning algorithms trained on a subset of citations to prioritise studies with the highest probability of meeting the review inclusion criteria (5). Once screening has commenced, when we reached the point that we had screened at least 200 records without finding a relevant study, we stopped manual assessment and assumed the remaining unread records were irrelevant. A custom classifier was not needed because the priority screening had an optimal performance. We identified all relevant studies by screening 5.3% (543 records) of the total number of retrieved records, 10,162. An estimate of almost 95% in workload saving.

10162 search results were imported to EPPI-Reviewer. No duplicates were identified within EPPI-Reviewer.

**Cochrane RCT classifier**

We run the Cochrane RCT classifier on the 10162 records in order to identify those with the highest probability of being an RCT. 3320 references were selected as *may be RCTs* and were therefore read at title and abstract. See Priority screening below. The 6841 references marked as *unlikely to be RCTs* were automatically discarded.

Figure B.1. Output of the Cochrane RCT classifier


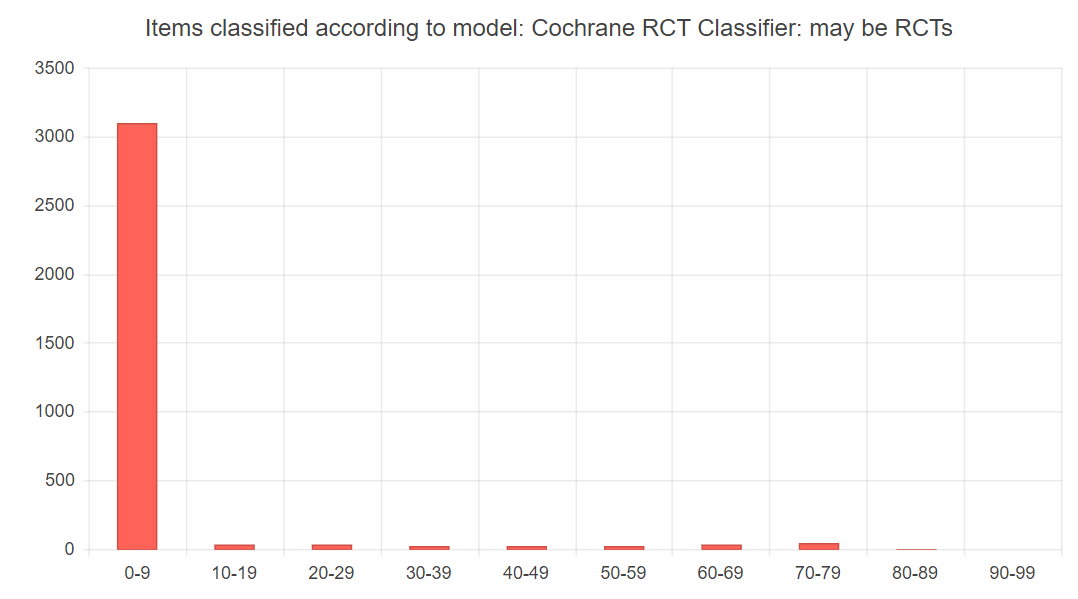


**Priority screening**

We started priority screening (also called the ranking algorithm) on the 3320 references from the Cochrane RCT classifier. We stopped title and abstract screening at reference 555 as we read 217 references without identifying any relevant RCTs.

Figure B.2. Output of the priority screening: screening curve


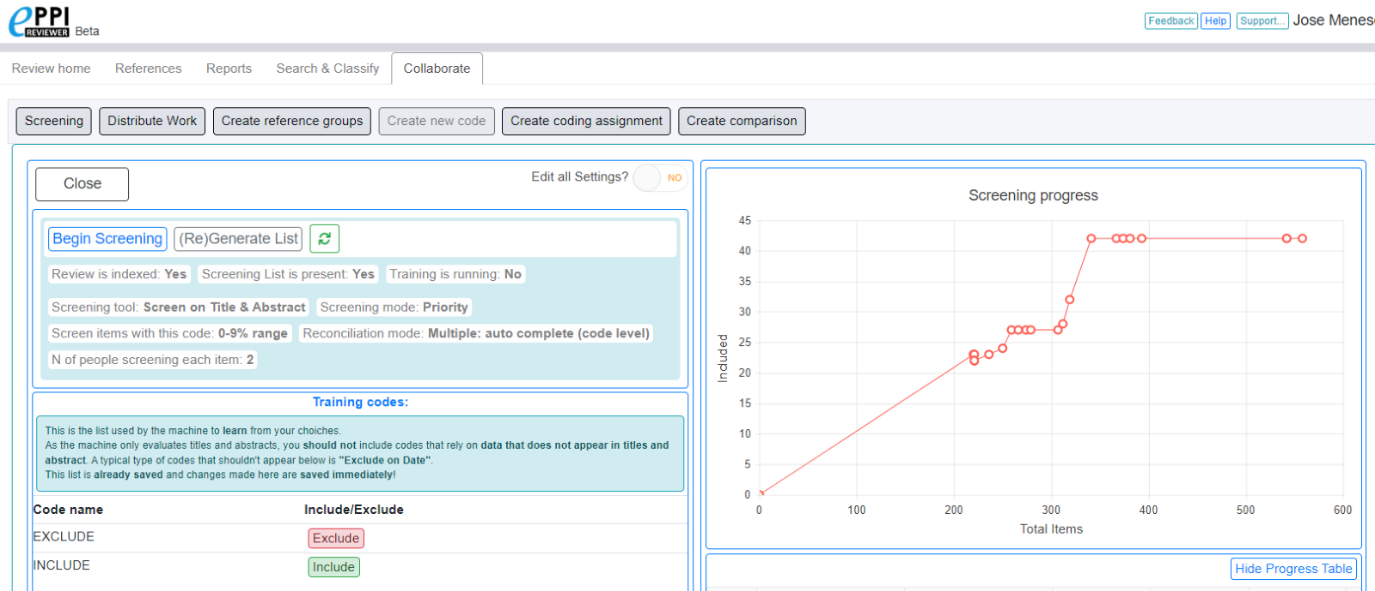


**Title and abstract (n=555) results**:

- Exclude 517 records
- Include 38 records

**Full-text screening** (n=39)

- EXCLUDE ineligible target group n=18
- EXCLUDE ineligible intervention n=1
- EXCLUDE ineligible study design n=8
- EXCLUDE ineligible outcomes n=1
- INCLUDE based on information in the full study n=10
- INCLUDE NCT record based on reference checking studies = 1

**Ten eligible (included) studies were identified from the EPPI-Reviewer first stage screening:**

1. Courser, M. W., Shamblen, S. R., Lavrakas, P. J., Collins, D., & Ditterline, P. (2009). The impact of active consent procedures on nonresponse and nonresponse error in youth survey data: Evidence from a new experiment. Evaluation Review, 33(4), 370-395. doi:10.1177/0193841x09337228.
2. Erhart, M., Wetzel, R. M., Krugel, A., & Ravens-Sieberer, U. (2009). Effects of phone versus mail survey methods on the measurement of health-related quality of life and emotional and behavioural problems in adolescents. BMC Public Health, 9, 491. doi:10.1186/1471-2458-9-491
3. Lygidakis, C., Rigon, S., Cambiaso, S., Bottoli, E., Cuozzo, F., Bonetti, S., . . . Marzo, C. (2010). A web-based versus paper questionnaire on alcohol and tobacco in adolescents. Telemedicine Journal & E-Health, 16(9), 925-930. doi:10.1089/tmj.2010.0062
4. Mauz, E., Hoffmann, R., Houben, R., Krause, L., Kamtsiuris, P., & Goswald, A. (2018). Mode equivalence of health indicators between data collection modes and mixed-mode survey designs in population-based health interview surveys for children and adolescents: Methodological study. Journal of Medical Internet Research Vol 20(3), 2018, ArtID e64, 20(3). doi:10.2196/jmir.7802
5. McMorris, B. J., Petrie, R. S., Catalano, R. F., Fleming, C. B., Haggerty, K. P., & Abbott, R. D. (2009). Use of web and in-person survey modes to gather data from young adults on sex and drug use: An evaluation of cost, time, and survey error based on a randomized mixed-mode design. Evaluation Review, 33(2), 138-158. doi:10.1177/0193841x08326463
6. Miech, R. A., Couper, M. P., Heeringa, S. G., & Patrick, M. E. (2021). The impact of survey mode on US national estimates of adolescent drug prevalence: Results from a randomized controlled study. Addiction, 116(5), 1144-1151. doi:10.1111/add.15249
7. Pejtersen, J. H. (2020). The effect of monetary incentive on survey response for vulnerable children and youths: A randomized controlled trial. PLoS ONE Vol 15(5), 2020, ArtID e0233025, 15(5). doi:10.1371/journal.pone.0233025
8. Raat, H., Mangunkusumo, R. T., Landgraf, J. M., Kloek, G., & Brug, J. (2007). Feasibility, reliability, and validity of adolescent health status measurement by the Child Health Questionnaire Child Form (CHQ-CF): internet administration compared with the standard paper version. Quality of life research, 16(4), 675‐685. doi:10.1007/s11136-006-9157-1
9. Trapl, E. S. (2007). Understanding adolescent survey responses: Impact of mode and other characteristics on data outcomes and quality. Dissertation Abstracts International: Section B: The Sciences and Engineering, 68(4-B), 2303.
10. Wettergren, L., Mattsson, E., & von Essen, L. (2011). Mode of administration only has a small effect on data quality and self-reported health status and emotional distress among Swedish adolescents and young adults. Journal of clinical nursing, 20(11‐12), 1568‐1577. doi:10.1111/j.1365-2702.2010.03481.x

Five further included studies (six publications) were identified from the citation searches:

1. Denniston/Eaton 2010
   1. Denniston, M. M., Brener, N. D., Kann, L., Eaton, D. K., McManus, T., Kyle, T. M., . . . Ross, J. G. (2010). Comparison of paper-and-pencil versus Web administration of the Youth Risk Behavior Survey (YRBS): Participation, data quality, and perceived privacy and anonymity. Computers in Human Behavior, 26(5), 1054-1060. doi:10.1016/j.chb.2010.03.006 (identified from the backwards citation search 20 June 2022)
   2. Eaton, D. K., Brener, N. D., Kann, L., Denniston, M. M., McManus, T., Kyle, T. M., . . . Ross, J. G. (2010). Comparison of paper-and-pencil versus web administration of the youth risk behavior survey (YRBS): Risk behavior prevalence estimates. Evaluation Review, 34(2), 137-153. doi:10.1177/0193841X10362491 (identified from the backwards citation search 20 June 2022)
2. Hamann, C., Schultze-Lutter, F., & Tarokh, L. (2016). Web-Based Assessment of Mental Well-Being in Early Adolescence: A Reliability Study. J Med Internet Res, 18(6), e138. doi:10.2196/jmir.5482 (identified from forward citation searching)
3. Raghupathy, S., & Hahn-Smith, S. (2013). The effect of survey mode on high school risk behavior data: A comparison between web and paper-based surveys. Current Issues in Education, 16(2). (identified from the backwards citation search 20 June 2022)
4. Van De Looij-Jansen, P. M., & De Wilde, E. J. (2008). Comparison of web-based versus paper-and-pencil self-administered questionnaire: effects on health indicators in Dutch adolescents. Health Services Research, 43(5 Pt 1), 1708-1721. doi:10.1111/j.1475-6773.2008.00860.x
5. Walser, S., & Killias, M. (2012). Who should supervise students during self-report interviews? A controlled experiment on response behavior in online questionnaires. Journal of Experimental Criminology, 8(1), 17-28. doi:10.1007/s11292-011-9129-5 (identified from the forwards citation search 2022-06-28)

One further study was identified from reading the included studies:

1. The effect of monetary incentive on survey response for vulnerable children and youth [NCT01741675]. (2022). Retrieved from https://clinicaltrials.gov/ct2/show/NCT01741675

**B.3 Excluded studies**

**Table B.5 Studies (n=28) retrieved by the database searches and excluded at full text assessment with reasons for exclusion**

| **Document reference** | **Reason(s) for exclusion** |
| --- | --- |
| Al Baghal, T. and P. Lynn (2015). "Using motivational statements in web-instrument design to reduce item-missing rates in a mixed-mode context." Public Opinion Quarterly 79(2): 568-579. | Target group: no information on adolescents |
| Bryan, L. N., Smith-Grant, J., Brener, N., Kilmer, G., Lo, A., Queen, B., & Underwood, J. M. (2022). Electronic Versus Paper and Pencil Survey Administration Mode Comparison: 2019 Youth Risk Behavior Survey. Journal of School Health, 92(8), 804-811. doi:10.1111/josh.13184 | Study design: not an RCT |
| Colasante, E., et al. (2019). "Paper-and-pencil versus computerized administration mode: Comparison of data quality and risk behavior prevalence estimates in the European school Survey Project on Alcohol and other Drugs (ESPAD)." PLoS ONE [Electronic Resource] 14(11): e0225140. | Study design: not an RCT |
| De Bruijne, M. and A. Wijnant (2013). "Comparing survey results obtained via mobile devices and computers: An experiment with a mobile web survey on a heterogeneous group of mobile devices versus a computer-assisted web survey." Social Science Computer Review 31(4): 482-504. | Study design: not an RCT |
| Dietz, N. A., et al. (2015). "Identifying misclassification in youth self-reported smoking status: testing different consent processes of biological sample collection to capture misclassification." Drug & Alcohol Dependence 149: 264-267. | Intervention: biological data collection with self-reported telephone survey data |
| Dolezal, C., et al. (2012). "A comparison of audio computer-assisted self-interviews to face-to-face interviews of sexual behavior among perinatally HIV-exposed youth." Archives of sexual behavior 41(2): 401-410. | Target group: subgroup of perinatally HIV-exposed youth |
| Duan, N., et al. (2007). "Survey conditioning in self-reported mental health service use: randomized comparison of alternative instrument formats." Health Services Research 42(2): 890-907. | Target group: participants’ age >18 |
| Fleming, C. B., et al. (2013). "Use of web and phone survey modes to gather data from adults about their young adult children: An evaluation based on a randomized design." Field Methods 25(4): 388-404. | Target group: parents survey vs telephone interview |
| Gagne, T., et al. (2014). "How important are paper copies of questionnaires? Testing invitations modes when studying social inequalities in smoking among young adults." International Journal of Public Health 59(1): 207-210. | Target group: participants aged 18-25 |
| Gilligan, C., et al. (2014). "Social networking versus Facebook advertising to recruit survey respondents: a quasi-experimental study." JMIR Research Protocols 3(3): e48. | Target group: participants aged 13-17. No mental health but general population |
| Hamilton, S., et al. (2011). "The effect of disclosure of mental illness by interviewers on reports of discrimination experienced by service users: a randomized study." International Review of Psychiatry 23(1): 47-54. | Target group: participants aged 18-65 |
| Hu SS, Gentzke A, Jamal A, Homa D, Neff L. (2020) “Feasibility of Administering an Electronic Version of the National Youth Tobacco Survey in a Classroom Setting”. Prev Chronic Dis. 17:E20. | No outcomes of interest |
| Huhtanen, P., et al. (2016). "Effects of telephone versus face-to-face survey modes on reports of alcohol-related attitudes, harms and alcohol consumption." Journal of Substance Use 21(4): 407-413. | Target group: results reported only for 15-29 age group |
| Lavender, J. M. and D. A. Anderson (2008). "A novel assessment of behaviors associated with body dissatisfaction and disordered eating." Body Image 5(4): 399-403. | Target group: participants aged 17-39; mean age 19yrs |
| Lim, M. S., et al. (2010). "Randomised controlled trial of paper, online and SMS diaries for collecting sexual behaviour information from young people." Journal of Epidemiology & Community Health 64(10): 885-889. | Target group: age is above high school, and participants do not have or provide information on mental health |
| Livingston, M. D., et al. (2015). "The effects of survey modality on adolescents' responses to alcohol use items." Alcoholism: Clinical & Experimental Research 39(4): 710-715. | Study design: not an RCT, longitudinal study |
| McLean, S. A., et al. (2014). "Prenotification but not envelope teaser increased response rates in a bulimia nervosa mental health literacy survey: A randomized controlled trial." Journal of Clinical Epidemiology 67(8): 870-876. | Target group: participants’ age >18 |
| Midanik, L. T. and T. K. Greenfield (2008). "Interactive voice response versus computer-assisted telephone interviewing (CATI) surveys and sensitive questions: the 2005 National Alcohol Survey." Journal of Studies on Alcohol & Drugs 69(4): 580-588. | Target group: adults aged 18 years and older |
| Midanik, L. T. and T. K. Greenfield (2010). "Reports of alcohol-related problems and alcohol dependence for demographic subgroups using interactive voice response versus telephone surveys: the 2005 US National Alcohol Survey." Drug & Alcohol Review 29(4): 392-398. | Target group: adults aged 18 years and older |
| Onoye, J. M., et al. (2012). "Use of incentives and web-based administration for surveying student alcohol and substance use in an ethnically diverse sample." Journal of Substance Use 17(1): 61-71. | Study design: not an RCT |
| Perri, P. F., et al. (2018). "A mixed-mode sensitive research on cannabis use and sexual addiction: Improving self-reporting by means of indirect questioning techniques." Quality & Quantity: International Journal of Methodology 52(4): 1593-1611. | Target group: university students |
| Randall, C. L., et al. (2013). "Collecting psychosocial self-report data in oral health research: impact of literacy level and computerised administration." Social Science & Dentistry 2(2): 80-87. | Target group: mean age 28 years |
| Richardson C G and Johnson J L; Ratner P A; Zumbo B D;. 2009. "The Influence of Web- Versus Paper-based Formats on the Assessment of Tobacco Dependence: Evaluating the Measurement Invariance of the Dimensions of Tobacco Dependence Scale". Substance Abuse 3:1-14. | Study design: not an RCT |
| Shaw, T., et al. (2015). "Bias in student survey findings from active parental consent procedures." British Educational Research Journal 41(2): 229-243. | Study design: parents were mailed a follow-up letter requesting passive consent for their child to participate in the study |
| Virtanen, V., et al. (2007). "Reducing nonresponse by SMS reminders in mail surveys." Social Science Computer Review 25(3): 384-395. | Target group: participants aged 15-34 years |
| Wetzels, W., et al. (2008). "Impact of prepaid incentives in face-to-face surveys: A large-scale experiment with postage stamps." International Journal of Public Opinion Research 20(4): 507-516. | Target group: participants aged 15-34 years |
| White, B., et al. (2007). "Self reported risk behaviour among injecting drug users: self versus assisted questionnaire completion." AIDS care 19(3): 441-447. | Study design: not an RCT |
| Wolford, G., et al. (2008). "A clinical trial comparing interviewer and computer-assisted assessment among clients with severe mental illness." Psychiatric Services 59(7): 769-775. | Target group: participants aged 18–65 years |
| Wyrick David L and Bond Lloyd. 2011. "Reducing sensitive survey response bias in research on adolescents: A comparison of web-based and paper-and-pencil administrations". American Journal of Health Promotion 25(5):349-352. | No outcomes of interest |

**EXCLUSIONS with reasons for Backward and Forward Citation**

**Table B.6 Studies retrieved by the backward and forward citation searches and excluded at full text assessment with reasons for exclusion**

| **Document reference** | **Reason(s) for exclusion** |
| --- | --- |
| Basnov, M., Kongsved, S. M., Bech, P., & Hjollund, N. H. (2009). Reliability of short form-36 in an Internet- and a pen-and-paper version. Informatics for Health and Social Care, 34(1), 53-58. doi:10.1080/17538150902779527 | Age: women referred to mammography |
| Bates, S. C., & Cox, J. M. (2008). The impact of computer versus paper-pencil survey, and individual versus group administration, on self-reports of sensitive behaviors. Computers in Human Behavior, 24, 903-916. doi:10.1016/j.chb.2007.02.021 | Age: unclear age of participants |
| Börkan, B. (2010). The mode effect in mixed-mode surveys: Mail and web surveys. Social Science Computer Review, 28(3), 371-380. doi:10.1177/0894439309350698 | Age: population Teachers |
| Brøgger, J., Nystad, W., Cappelen, I., & Bakke, P. (2007). No increase in response rate by adding a web response option to a postal population survey: A randomized trial. Journal of medical Internet research, 9(5). doi:10.2196/jmir.9.5.e40 | Age: population 20-40yrs |
| Callas, P. W., Solomon, L. J., Hughes, J. R., & Livingston, A. E. (2010). The influence of response mode on study results: Offering cigarette smokers a choice of postal or online completion of a survey. Journal of medical Internet research, 12(4), e46p.41-e46p.48. doi:10.2196/jmir.1414 | Age: adults |
| Guo, Y., Kopec, J. A., Cibere, J., Li, L. C., & Goldsmith, C. H. (2016). Population survey features and response rates: A randomized experiment. American journal of public health, 106(8), 1422-1426. doi:10.2105/AJPH.2016.303198 | Age: Household adults (aged 18 years and older) |
| Hu SS, Gentzke A, Jamal A, Homa D, Neff L. (2020) “Feasibility of Administering an Electronic Version of the National Youth Tobacco Survey in a Classroom Setting”. Prev Chronic Dis. 17:E20 | Outcomes: no outcomes of interest |
| Khazaal, Y., Chatton, A., Monney, G., Nallet, A., Khan, R., Zullino, D. F., & Etter, J. (2015). Internal consistency and measurement equivalence of the cannabis screening questions on the paper-and-pencil face-to-face ASSIST versus the online instrument. Substance abuse treatment, prevention, and policy, 10, 8-8. doi:10.1186/s13011-015-0002-9 | Age: mean age of the participants was 41 ±11.5 years. |
| Kongsved, S. M., Basnov, M., Holm-Christensen, K., & Hjollund, N. H. (2007). Response rate and completeness of questionnaires: A randomized study of internet versus paper-and-pencil versions. Journal of medical Internet research, 9(3). doi:10.2196/jmir.9.3.e25 | Age: Women referred to mammography |
| Kurth BM, Kamtsiuris P, Hölling H, Schlaud M, Dölle R, Ellert U, et al. The challenge of comprehensively mapping children's health in a nation-wide health survey: Design of the German KiGGS-Study. BMC Public Health 2008;8. DOI: 10.1186/1471-2458-8-196 | Design: no comparison group |
| Lampert, T., & Kuntz, B. (2014). Tobacco and alcohol consumption among 11- to 17-year-old adolescents: Results of the KiGGS study: First follow-up (KiGGS Wave 1). Bundesgesundheitsblatt - Gesundheitsforschung - Gesundheitsschutz, 57(7), 830-839. doi:10.1007/s00103-014-1982-8 | Language |
| Lucia, S., Herrmann, L., & Killias, M. (2007). How important are interview methods and questionnaire designs in research on self-reported juvenile delinquency? An experimental comparison of Internet vs paper-and-pencil questionnaires and different definitions of the reference period. Journal of Experimental Criminology, 3(1), 39-64. doi:10.1007/s11292-007-9025-1 | No mental health *delinquency |
| Mavletova, A., & Couper, M. P. (2013). Sensitive Topics in PC Web and Mobile Web Surveys: Is There a Difference? Survey Research Methods, 7, 191-205. doi:10.18148/srm/2013.v7i3.5458 | Country: not High Income |
| Mayr, A., Gefeller, O., Prokosch, H., Pirkl, A., Fröhlich, A., & de Zwaan, M. (2012). Web-based data collection yielded an additional response bias--but had no direct effect on outcome scales. Journal of Clinical Epidemiology, 65, 970-977. doi:10.1016/j.jclinepi.2012.03.005 | Age: Mean age 47yrs |
| Quijada, Y., Saldivia, S., Bustos, C., Preti, A., Ochoa, S., Castro-Alzate, E., & Siddi, S. (2022). Measurement invariance between online and paper-and-pencil formats of the Launay-Slade Hallucinations scale-extended (LSHS-E) in the Chilean population: Invariance between LSHS-E formats. Current psychology (New Brunswick, N.J.), 1-undefined. doi:10.1007/s12144-021-02497-7 | Design: No information on randomization; mean age 21years |
| Raghupathy, S., & Hahn-Smith, S. (2012). Reliability of the High School Youth Risk Behavior Survey When Administered Online. International quarterly of community health education, 32, 135-148. doi:10.2190/iq.32.2.d | Design (web performance) |
| Richards, J., Wiese, C., Katon, W., Rockhill, C., McCarty, C., Grossman, D. C., . . . Richardson, L. P. (2010). Surveying adolescents enrolled in a regional health care delivery organization: mail and phone follow-up--what works at what cost? Journal of the American Board of Family Medicine: JABFM, 23, 534-541. doi:10.3122/jabfm.2010.04.100019 | Design: Randomly selected by not randomly allocated to an intervention |
| Sakshaug, J. W., Cernat, A., & Raghunathan, T. E. (2019). Do Sequential Mixed-Mode Surveys Decrease Nonresponse Bias, Measurement Error Bias, and Total Bias? An Experimental Study. Journal of Survey Statistics and Methodology, 7, 545-571. doi:10.1093/jssam/smy024 | Age: “young adult drivers” |
| Shih, T. H., & Xitao, F. (2008). Comparing response rates from web and mail surveys: A meta-analysis. Field Methods, 20(3), 249-271. doi:10.1177/1525822X08317085 | Design meta-analysis |
| Shim, J. M., Shin, E., & Johnson, T. P. (2013). Self-rated health assessed by web versus mail modes in a mixed mode survey: The digital divide effect and the genuine survey mode effect. Medical Care, 51(9), 774-781. doi:10.1097/MLR.0b013e31829a4f92 | Age: adults |
| Thompson, L. A., Mercado, R., Martinko, T., & Acharya, R. (2018). Novel Interventions and Assessments Using Patient Portals in Adolescent Research: Confidential Survey Study. Journal of Medical Internet Research, 20, e101-undefined. doi:10.2196/jmir.8340 | Design: not randomized groups |
| Ward, P., Clark, T., Zabriskie, R. B., & Morris, T. (2014). Paper/Pencil Versus Online Data Collection. Journal of Leisure Research, 46, 84-105. doi:10.1080/00222216.2014.11950314 | Age: unclear “had never married, with 13% married and the rest either divorced or other” |
| Wu, Y., & Newfield, S. A. (2007). Comparing Data Collected by Computerized and Written Surveys for Adolescence Health Research. Journal of School Health, 77(1), 23-28. doi:https://doi.org/10.1111/j.1746-1561.2007.00158.x | Design: not randomized |
| Zhang, X., Kuchinke, L., Woud, M. L., Velten, J., & Margraf, J. (2017). Survey method matters: Online/offline questionnaires and face-to-face or telephone interviews differ. Computers in Human Behavior, 71, 172-180. doi:10.1016/j.chb.2017.02.006 | Age: adults |

**References to the appendix**

**References**

1. Lefebvre C, Glanville J, Briscoe S, Featherstone R, Littlewood A, Marshall C, Metzendorf M-I, Noel-Storr A, Paynter R, Rader T, Thomas J, Wieland LS. Chapter 4: Searching for and selecting studies. In: Higgins JPT, Thomas J, Chandler J, Cumpston M, Li T, Page MJ, Welch VA (editors). Cochrane Handbook for Systematic Reviews of Interventions version 6.3 (updated February 2022). Cochrane, 2022. Available from [www.training.cochrane.org/handbook](http://www.training.cochrane.org/handbook).

2. The effect of monetary incentive on survey response for vulnerable children and youth [NCT01741675]. (2022). Retrieved from https://clinicaltrials.gov/ct2/show/NCT01741675

3. The EndNote team. (2013). EndNote 20.2.1. Clarivate Analytics. Philadelphia, PA. Retrieved from https://endnote.com

4. Thomas, J., Graziosi, S., Brunton, J., Ghouze, Z., O'Driscoll, P., Bond, M., & et al. (2010 ). EPPI-Reviewer 4. Retrieved from https://eppi.ioe.ac.uk/cms/Default.aspx?tabid=2967. from EPPI-Centre, Social Science Research Unit, UCL Institute of Education https://eppi.ioe.ac.uk/cms/Default.aspx?tabid=2967

5. Thomas, J., McDonald, S., Noel-Storr, A., Shemilt, I., Elliott, J., Mavergames, C., & Marshall, I. J. (2021). Machine learning reduced workload with minimal risk of missing studies: development and evaluation of a randomized controlled trial classifier for Cochrane Reviews. Journal of Clinical Epidemiology, 133, 140-151. doi:10.1016/j.jclinepi.2020.11.003
