# Supplementary material for: Methods, strategies, and incentives to increase response to mental health surveys among adolescents: a systematic review
Source: BMC Med Res Methodol. 2023 Nov 16;23:270. doi: 10.1186/s12874-023-02096-z (PMC10652438; doi:10.1186/s12874-023-02096-z)
Supplement: Supplementary file 3 — Additional file 3. Detailed data extraction for the included studies. [file 12874_2023_2096_MOESM3_ESM.docx]

**Appendix C. Detailed data extraction tables**

**C.1 Comparison 1: Web vs paper-and-pencil administration (n=9)**

**Denniston 2010/Eaton 2010**

| Inclusion/exclusion criteria | Adolescents, either 9th- or 10th-grade students |
| --- | --- |
| Objective | 1) To determine the feasibility and acceptability of allowing students to complete a Web survey in their own time; (2) To assess the extent to which data quality and respondents’ perceived privacy and anonymity vary by mode; and (3) To understand the effect of programmed skip patterns (where students can choose to skip questions) on data quality. |
| Trial date | NR |
| Data (participants) | Age: ≤14 years (21.8%), 15 years (46%); 16 years (24.6%); ≥17 years (7.6%)  Male (%): 48%  9th- or 10th-grade students  White non-Hispanic (%): 44% on average  Black non-Hispanic (%): 27% on average  Hispanic (%): 20% on average |
| Parents involved | Local parental permission procedures were followed in each school. |
| Comparisons | Mode 1: PAPI (n=1729)  Mode 2: Web (n=4057)  2.1 Survey completed in-class Web without skips – used school’s stationary computer lab (n=1735)  2.2. Survey completed In-class Web with skips – used school’s stationary computer lab (n=1763)  2.3. On student’s own Web (n=559) |
| Strategy | Focus on mode of administration.  In all conditions, participation was voluntary and anonymous. |
| Incentive | NR |
| Field of study | Behaviours (consumption of alcohol, tobacco, and other drugs) |
| Survey(s) | Youth Risk Behavior Surveys (YRBS) relevant survey questions:   - Sadness - felt sad or hopeless almost every day for 2 or more weeks in a row - Suicide attempt - attempted suicide during the last 12 months |
| Recruitment strategy | Convenience sample of schools that had the capability to accommodate Web administration of the Youth Risk Behavior Surveillance System and were willing to participate. |
| Data management, number of participants withdrawn or not responding | Of the 7747 students, 220 were absent the day of the survey, 51 refused participation, 132 had participation refused by parents, 240 returned no consent form, 1195 did not return a survey, and 123 returned a survey that failed data edits. |
| Funding and Conflicts of interests | NR |

NR- not reported; PAPI – paper and pencil intervention

**Hamann 2016**

| Inclusion/exclusion criteria | NR |
| --- | --- |
| Objective | To examine the accuracy of responses from web-based questionnaires with paper-and-pencil versions of depression and anxiety questionnaires |
| Trial date | NR |
| Data (participants) | Mean age (SD): total mean 12.78 (0.78) years, ranging from 11 to 13 years |
| Parents involved | Parents were instructed to leave the child alone to fill out the web-based and paper-and-pencil forms. |
| Comparisons | 1. PAPI: participants received the questionnaires by mail and a self-addressed stamped envelope was provided for returning the questionnaires.  2. Web: participants received an identification number, a password, and a link to the survey website. |
| Strategy | NR |
| Incentive | Participants received gift vouchers for taking part in the study. |
| Field of study | Adolescents’ mental health |
| Surveys(s) | German version of Children’s Depression Inventory (CDI) and the Spence Children’s Anxiety Scale (SCAS)  In both tools, higher scores represent worse anxiety/depression. |
| Recruitment strategy | NR |
| Data management, number of participants withdrawn or not responding | NR |
| Funding and Conflicts of interests | NR |

NR- not reported; PAPI – paper and pencil intervention; SD – standard deviation

**Lygidakis 2010**

| Inclusion/exclusion criteria | Adolescents with disabilities that could prevent them from participating in the study were excluded. |
| --- | --- |
| Objective | To compare health behaviour data by mode of survey delivery and assessing the feasibility of the application. |
| Trial date |  |
| Data (participants) | Adolescents aged 14 to16 years, in senior high schools  Males (%): 50%  Italian, Caucasian  Mostly medium family income index |
| Parents involved | Because of the anonymous way data were collected, no parental consent was requested. |
| Comparisons | 1. PAPI (n=97), completed in a supervised classroom.  2. Web (n=93), completed in a supervised classroom; students were asked to access web application directly, fill out, and submit their own questionnaire. |
| Strategy | Reassurance of anonymity – teachers did not intervene |
| Incentive | NR |
| Survey(s) | Ad hoc questionnaire based on the European School Survey Project on Alcohol and Other Drugs questionnaire (2003) and the fifth Doxa national survey |
| Field of study | Behaviours (alcohol and tobacco) |
| Recruitment strategy | NR |
| Data management, number of participants withdrawn or not responding | Data collection was carried out with the Lumos Platform.  18 respondents were excluded from the study after randomization. Partially compiled questionnaires were allowed. |
| Funding and Conflicts of interests | Funding NR  No competing financial interests. |

NR- not reported; PAPI – paper and pencil intervention

**Mauz 2018**

| Inclusion/exclusion criteria | Children and adolescents registered in local resident registries. |
| --- | --- |
| Objective | To determine “whether the prevalence rates or mean values of self- and parent-reported health indicators for children and adolescents aged 0-17 years differ between self-administered paper-based questionnaires (SAQ-paper) and self-administered Web-based questionnaires (SAQ-Web), as well as between a single-mode control group and different  mixed-mode groups.” |
| Trial date | NR |
| Data (participants) | PAPI: 11-13 years (44%); ≥14 years (56%)  Web: 11-13 years (44.8%); ≥14 years (55.2%)  Males (%): PAPI (45.4%); Web (55.2%)  “Migration background” (no further detail reported): PAPI (11.8%); Web (13.7%)  Net household income:  Middle (2nd–4th quartile): PAPI (57%); Web (56.1%)  Low (1st quartile): PAPI (18.4%); Web (14.3%)  Participants were from rural and urban areas. |
| Parents involved | Parents completed the surveys for children aged 0-11 years and adolescents (aged 12-17 years) completed surveys themselves. |
| Comparisons | Sequential mixed-mode design (web and paper self-administered questionnaires (SAQ)): respondents were sent an invitation letter and an online access code, followed 3 weeks later with a reminder letter and a paper-based questionnaire.  Concurrent mixed-mode design (web and paper SAQ): respondents were sent an invitation letter, a paper-based questionnaire, and an online access code.  Preselect mixed-mode design (web and paper SAQ): respondents were sent  the invitation along with a postcard asking participants to choose one of the 2 options (SAQ-Web or SAQ-paper), followed by a reminder with the same offer.  Single-mode design, paper SAQ (control): respondents were sent an invitation letter and paper-and-pencil questionnaires, followed by a reminder after 3 weeks. |
| Strategy | Reminder emails and phone calls |
| Incentive | The parents who completed questionnaires on behalf of young children received a shopping voucher to the value of €10 euros. Adolescents who completed questionnaires themselves received a shopping voucher to the value of €10 euros. |
| Survey(s) | The German Health Interview and Examination Survey for Children and Adolescents (KiGGS) and The Strengths and Difficulties Questionnaire (SDQ). |
| Field of study | Focus on survey administration mode. |
| Recruitment strategy | Individuals were invited by mail to participate in the study and received instructions according to assigned survey mode. |
| Data management, number of participants withdrawn or not responding | 4032 out of 11148 participants completed the study. |
| Funding and Conflicts of interests | This project was supported by the Robert Koch Institute and the German Federal Ministry of Health within the German Health Monitoring System.  Conflicts of interests: none declared. |

PAPI – paper and pencil intervention; SAQ - self-administered questionnaires

**Miech 2021**

| Inclusion/exclusion criteria | This study excluded students who were unable to complete self-reported questionnaires as a result of low English proficiency or physical, sensory, or reading disabilities. The sample sizes reported (20985 students who recorded their survey answers on tablets and 20881 who recorded their answers using PAPI) excluded 1.5% of students who did not answer any drug use questions. |
| --- | --- |
| Objective | This study tested whether (a) overall, self‐reported drug use estimate variations by mode of survey delivery is higher when using electronic tablets versus paper‐and‐pencil surveys, (b) socio‐demographics moderate survey mode effects and (c) levels of missing data are lower for electronic tablet versus paper‐and‐pencil modes |
| Trial date | 2019 |
| Data (participants) | Students in 8th grade (51%), 10th grade (50%), 12th grade (53%)  Males (%): 51%  White non-Hispanic 49.3%, non-white 51.9% |
| Parents involved | Informed consent: passive or active (written), per school policy was obtained from parents for students younger than 18 years and from students aged 18 years or older. |
| Comparisons | Mode 1: PAPI  Mode 2: Web. Responses were encrypted and transmitted to the University of Michigan at the first internet connection opportunity, typically immediately after survey completion. University staff provided technical assistance for tablet users. |
| Strategy | The questionnaire took place in classrooms during normal class periods; however, circumstances in some schools required the use of “larger group administrations” (no further details provided).  Students were instructed to skip any questions they did not feel comfortable answering and could change answers at any point (by erasing responses on paper‐and pencil surveys or going back and overriding answers on tablets). |
| Incentive | NR |
| Survey(s) | Monitoring the Future (MTF) school-based national survey |
| Field of study | Behaviours (drug estimate variations by mode of survey delivery) |
| Recruitment strategy | Voluntary school and student participation. |
| Data management, number of participants withdrawn or not responding | University personnel administered the questionnaires in each school.  Levels of missing data were lower for electronic tables (Web). |
| Funding and Conflicts of interests | Funded by the National Institute on Drug Abuse, part of the US National Institutes of Health, by grant no. DA001411.  Conflicts of interests: none |

NR- not reported; PAPI – paper and pencil intervention

**Raat 2007**

| Inclusion/exclusion criteria | NR |
| --- | --- |
| Objective | To compare paper and pencil with internet (Web) mode for Child Health Questionnaire Child Form (CHQ-CF) administration with regard to the following indicators:  (a) the number of missing answers (indicator of feasibility), (b) the distribution of the scale scores including mean scale scores in the whole sample and in gender and age specific subgroups, (c) the internal consistency reliability of multi-item scales (indicator of reliability), (d) the ability of the CHQ-CF to discriminate between subgroups with and without self-reported chronic conditions (indicator of construct validity). |
| Trial date | 2003 |
| Data (participants) | Mean age (SD): 14.7 years (0.68), range 13 to 17 years  Male (%): 46%  Participants from rural and urban areas  Born in the Netherlands: 93%  Depression or anxiety attacks: PAPI (36.8%) vs Web (38.8%)  The prevalence of self-reported chronic conditions (e.g., asthma, allergies, visual problems, chronic lower back pain) ranged from 7% to 25%. These conditions were equally distributed in the two survey mode groups. |
| Parents involved | The parents and students each received written information about the study several weeks before data collection; parents could refuse their child’s participation, and participation by the students was voluntary. |
| Comparisons | Mode 1: PAPI (n=458)  Mode 2: Web (n=475) used the same wording of the items and instructions from the conventional paper format and was developed through a generic internet tool using PHP (4.0.1), MySQL (3.22), and JavaScript (1.3). The web version of the questionnaire listed the items of each CHQ-CF scale on a separate webpage. The internet version did not allow the respondent to select more than one answer to each item of the CHQCF and it checked the questionnaire for missing answers before the respondent could log out. If one or more of the items were not answered, the internet version prompted the respondent to go back to complete those items; but, if the user failed to log out properly, missing answers would remain. |
| Strategy | Students completed the questionnaires, either on paper or online in a classroom with computers linked to the internet, under the supervision of a research assistant; the students were allowed adequate privacy. |
| Incentive | None |
| Surveys(s) | Child Health Questionnaire Child Form (CHQ-CF) |
| Field of study | Number of answers missing from the survey  CHQ-CF scores by mode of survey administration  CHQ-CF scores in the whole sample and in gender and age specific subgroups  Ability of CHQ-CF to discriminate between subgroups with and without self-reported chronic conditions |
| Recruitment strategy | 55 classes of various educational levels in the 3rd year of seven secondary schools (participants aged 13–17 years old) in Vlaardingen (metropolitan area) and Harderwijk (rural area), The Netherlands. The study does not report if these were all of the schools in the areas or whether this is a sample. |
| Data management, number of participants withdrawn or not responding | NR |
| Funding and Conflicts of interests | Netherlands Organization for Health Research and Development (ZonMw) Prevention Research Program Grant #2100.0066.  Conflicts of interests: NR |

NR- not reported; PAPI – paper and pencil intervention

**Raghupathy 2013**

| Inclusion/exclusion criteria | NR |
| --- | --- |
| Objective | To determine whether there were significant differences in the estimate variations by mode of survey delivery on high-risk behaviours and attitudes regarding alcohol use depending on whether Youth Risk Behavior Surveys (YRBS) questions were presented on a computer versus on paper. |
| Trial date | NR |
| Data (participants) | High school students in grades 9-12. Almost two-thirds of students (63.6%) were in either 9th or 10th grade.  Male (%): 50.4%  White non-Hispanic (%): 60% on average  Native Americans (%): 30% on average  Predominantly rural area. About half the students were eligible for free or reduced priced school lunches. |
| Parents involved | Active consent was sought from parents, while students submitted assent forms. |
| Comparisons | Mode 1: PAPI (n=181)  Mode 2: Web (n=160), incorporated programming features that promoted response confidentiality (enhanced privacy). The survey layout was designed to show just one question at a time on the computer screen. Once a student entered a response, the survey automatically progressed to the next question thereby instantly refreshing the screen. *This auto progression feature also eliminated non-responses because the students could only move on to the next question after the previous one was answered*. Online respondents were also automatically logged out after the last question. |
| Strategy | Paper and web surveys were administered in classrooms. Teachers were responsible for parental notifications at least two weeks before the study and for devising the schedules for computer lab use.  Data were deposited on an external server ensuring that teachers did not have access to the data. |
| Incentive | NR |
| Survey(s) | Youth Risk Behavior Surveys (YRBS) |
| Field of study | Behaviours (alcohol) |
| Recruitment strategy | Convenience sample, otherwise unclear |
| Data management, number of participants withdrawn or not responding | NR |
| Funding and Conflicts of interests | NR |

NR- not reported; PAPI – paper and pencil intervention

**Trapl 2007**

| Inclusion/exclusion criteria | Inclusion: students with different levels of literacy skills, including sentence comprehension, listening comprehension, and English mastery as measured by the Language/US index.  Exclusion: students with extreme cognitive, behavioural, and physical disabilities as identified by their special need teachers (n=2) or with limited English proficiency (n=11) were excluded from the study. |
| --- | --- |
| Objective | To examine the differential effects of three different data collection modes (SAQ, PDA, APDA) on the number of questions answered, data quality, and the student’s evaluation of the process.  SAQ - self-administered questionnaires  PDA – personal digital assistant  APDA – Audio-enhanced personal digital assistant |
| Trial date | 2006 |
| Data (participants) | Mean age (SD): 13.1 (0.75) years  Male (%): 52%  Caucasian (%): 23% on average  African American (%): 29.6% on average  Hispanic (%): 37.6% on average  Lived with 2 parents (50%)  Students with a range of abilities, including students with cognitive, behavioural and/or health deficits. |
| Parents involved | Active parental consent and student assent were required for participation in the study. Although written parental consent was the ideal, the Institutional Review Board also approved an alternative approach to recruitment that allowed the research team to obtain parental consent by phone or e-mail and subsequently confirm the consent decision by a letter to each student’s home. |
| Comparisons | Mode 1: PAPI (n= 90)  Mode 2: Web (n= 185)  2.1 PDA (n= 93)  2.2 APDA (n= 92) |
| Strategy | Following survey completion, students completed a paper-based debriefing survey, assessing students’ survey experience. Academic and behavioral assessments were completed by each student’s teacher. |
| Incentive | None |
| Survey(s) | Ad-hoc questionnaire, 178-question survey |
| Field of study | Behaviour (alcohol and marijuana)  To examine whether three different data collection modes (SAQ, PDA, APDA) were impacted by level of reading proficiency, language or attention issues |
| Recruitment strategy | Participants were stratified based on GRADE scores, as follows:  Prior to randomization into the study, students completed the Sentence Comprehension and Listening Comprehension sections of the Group Reading and Diagnostic Evaluation (GRADE). Students were stratified into one of three groups based on a combination of their stanine scores on the Sentence Comprehension (SC) and Listening Comprehension (LC) sections of the GRADE: (1) moderate/high SC and moderate/high LC; (2) moderate/high SC and low LC or low SC and moderate/high LC; and (3) low SC and low LC. Following this stratification, students within each of these strata in each school were individually randomized to one of the three data collection modes (SAQ, PDA, or APDA). |
| Data management, number of participants withdrawn or not responding | NR |
| Funding and Conflicts of interests | Supported through funds from the National Institute of Child Health and Human Development: HD-R01-41364-S2  Conflict of interests: NR |

NR- not reported

**van de Looij-Jansen 2008**

| Inclusion/exclusion criteria | NR |
| --- | --- |
| Objective | To investigate differences in responses related to (mental) health and behaviour between two methods of data collection: web-based and paper and pencil. |
| Trial date | 2005 |
| Data (participants) | Participants’ ages ranged between 14 to 16 years  Male (%): PAPI (48%); Web (56%)  Dutch background: PAPI (46.7%); Web (41.9%)  Turkish background: PAPI (9.7%); Web (13.3%)  Moroccan background: PAPI (10.8%); Web (12.2%)  Surinamese/Dutch Antillean/Aruban: PAPI (12.7%); Web (14.4%)  Various educational levels (basic pre-vocational, theoretical pre-vocational, general secondary, pre-university education) |
| Parents involved | Parents received an information letter and were given the opportunity to refuse their child’s participation – consent is legally required for this type of research. |
| Comparisons | Mode 1: PAPI (n= 261), students were given a questionnaire with their name printed on the front page. It was filled out in the classroom.  Mode 2: Web (n= 270), students completed the questionnaire in the school computer lab.  The two versions of the questionnaire were identical in terms of the questions asked, their wording, and their order of presentation in the survey. |
| Strategy | The study was part of the Youth Health Monitor Surveillance system Rotterdam (YMR), which is incorporated in the regular check-ups of the preventive youth health care system.  The questionnaires were the Dutch self-report version of the Strengths and Difficulties Questionnaire (SDQ) and the Child Health Questionnaire (CHQ). |
| Incentive | NR |
| Survey(s) | The Dutch self-report version of the Strengths and Difficulties Questionnaire (SDQ): sum score 0–10; higher scores mean worse mental health  The Child Health Questionnaire (CHQ) |
| Field of study | Mental health (psychological well-being, suicidal ideation, and aggressive behaviour), and behaviour (smoking, alcohol, marihuana, and sexual behaviour) |
| Recruitment strategy | Poorly reported. Five secondary schools at various educational levels were approached (and all agreed) for participation in this study. |
| Data management, number of participants withdrawn or not responding | Missing data were present in <5 percent of survey responses and were excluded. Reasons for absence were mainly illness. |
| Funding and Conflicts of interests | NR |

NR- not reported

**Comparison 2: Telephone interview vs postal questionnaires**

**Erhart 2009**

| Inclusion/exclusion criteria | Adults living in Germany with children aged 8 to 17 years. Four thousand families were selected on the basis of region, population, and a cost-of-living classification. |
| --- | --- |
| Objective | To study differences in using telephone and mail survey methods to measure health-related quality of life and emotional and behavioural problems in children and adolescents. |
| Trial date | 2004 |
| Data (participants) | Mean age (SD):  Telephone: 13.32 years (2.88)  Postal/mail survey: 13.38 years (2.79)  Male (%):  Telephone mode: 49%  Postal/mail survey: 51%  Samples were comparable with regards to the basic socio-demographic and socio-economic variables. |
| Parents involved | The parents and children in a family were interviewed separately from one another. Informed consent was collected from the parents. |
| Comparisons | Mode 1: Telephone (n=825) (see *strategy* below)  Mode 2: Postal/mail survey (n=912),  Households were provided with a stamped envelope for returning the questionnaire. After two and four weeks, identical reminders were sent to households that had not replied. |
| Strategy | Computer-assisted telephone interviews (CATI) were conducted mainly between 3 and 8 p.m. to increase the chances of reaching working adults. Specially trained interviewers contacted the households 1-2 weeks after the letter of information was sent out or after receiving the telephone number. Up to 12 attempts were made to contact the respondents. The parents and children in a family were interviewed separately from one another. The interviewers read aloud the time frame of the item, the item statement, and the item answer categories for every item. |
| Incentive | NR |
| Survey(s) | KINDL-R (Children’s, and adolescents’ health-related quality of life)  Strengths and Difficulties Questionnaire (SDQ)  Total score, higher scores indicate worse mental health |
| Field of study | Mental health and behaviour (health-related quality of life, emotional and behavioural problems in children and adolescents) |
| Recruitment strategy | Data were collected in the context of a nation-wide survey on the equivalence of aspects of health between children and their parents. |
| Data management, number of participants withdrawn or not responding | Cases with missing values were excluded for each separate analysis (pairwise), the numbers reported in the results differ from those reported in the sample description. |
| Funding and Conflicts of interests | The study was funded by a grant from the German Federal Robert Koch-Institut.  Conflict of interest: none. |

SD – standard deviation

**Wettergren 2011**

| Inclusion/exclusion criteria | Civil registered adolescents and young adults |
| --- | --- |
| Objective | The study investigated whether there are interaction effects for survey mode, age and gender on adolescents’ and young adults’ self-reports of health status and emotional distress. Additionally, it was investigated whether there is an effect of mode of administration on response rate and data quality (i.e., internal consistency, completeness of data and floor and ceiling effects on adolescents’ and young adults’ self-reports of health status and emotional distress). |
| Trial date | 2005 |
| Data (participants) | 13-15 years:  Telephone mode n = 89; Postal/mail survey n = 89–91  16-19 years:  Telephone mode n = 119; Postal/mail survey n = 108–110.  20-23 years:  Telephone mode n = 92; Postal/mail survey n = 83–84.  Male (%):  Telephone mode 49%; Postal/mail survey 46% |
| Parents involved | Parents’ consent was sought for those adolescents younger than 18 years. |
| Comparisons | Mode 1: Telephone (n=300), participants received a letter with information about the study, followed up after one week. Five interviewers performed the telephone interviews.  Mode 2: Postal/mail survey/PAPI (n=285), participants received a letter containing the Short Form 36 (SF-36) and the Hospital Anxiety and Depression Scale (HADS) and written information, and a stamped and addressed envelope. |
| Strategy | Reminders were sent to those who did not return the instruments within two weeks. |
| Incentive | All participants received a cinema ticket by mail. |
| Survey(s) | Short Form 36 (SF-36) and the Hospital Anxiety and Depression Scale (HADS) |
| Field of study | Mental health (health status, emotional distress, anxiety and depression) |
| Recruitment strategy | “Statistics Sweden selected the sample from a register of the total Swedish population using “a stratified quota sampling scheme”. 840 persons were invited to participate, 391 in a telephone interview and 449 with a postal questionnaire. |
| Data management, number of participants withdrawn or not responding | Reasons for non-participation were only recorded for the telephone interview. Reasons were illness or language difficulties (2.1%), impossible to reach (9.2%) and not wishing to participate (11.3%). |
| Funding and Conflicts of interests | Support from the Swedish Cancer Society and The Children’s Swedish Cancer Foundation.  Conflicts of interest: NR |

NR – not reported; PAPI – paper and pencil intervention

**Comparison 3: Active vs Passive parental consent**

**Courser 2009**

| Inclusion/exclusion criteria | School districts that participated in the biennial survey (Kentucky Incentives for Prevention survey) between 1999 and 2006. |
| --- | --- |
| Objective | To investigate the impact of consent procedures on participation rates, demographic characteristics of the survey sample, and estimates of alcohol, tobacco, and other drug use. |
| Trial date | Data were collected in 2007. |
| Data (participants) | Mean age (SD):  Active 13.5 (2.1) years; Passive 14 (2.2) years  Males (%):  Active (44%); Passive (52%)  Children in poverty (%): 28% on average  Rural (%): Active (78%); Passive (84%) |
| Parents involved | Parents needed to sign consent form. |
| Comparisons | 1. Active parental consent  2. Passive parental consent |
| Strategy | Students received at least one reminder to return the consent form to the survey administrator. |
| Incentive | School districts received a US$500 cash incentive if selected to participate in the study and if they adhered to the study protocol. Students who returned a consent form (signed or unsigned by parents) entered into a draw for one or more gift cards to local merchants or coupons for free soft drinks at school activities. Monetary incentives were also offered to the passive consent group but was declined. |
| Survey(s) | Survey used: Kentucky Youth Outcomes Survey (KYOS) |
| Field of study | Behaviours (alcohol, tobacco, and other drugs) |
| Recruitment strategy | Three stages: 1) solicited district interest to participate with incentive of U$500; 2) districts were paired according to sociodemographic characteristics (yoked design); and 3) seven of the best-matching pairs of school districts were selected plus other districts allocated using random assignment. |
| Data management, number of participants withdrawn or not responding | Three of the 14 school districts notified that they were unable to fulfill their commitment to the project (2 active, 1 passive); they were replaced with another three schools. 47 participants (0.6%) were eliminated due to poor quality data on their responses. |
| Funding and Conflicts of interests | The National Institutes on Drug Abuse, grant #1 R01DA019972-01A1, M. Courser, PI.  Conflicts of interests: not reported |

SD - standard deviation

**Comparison 4: Web first vs in-person first interview**

**McMorris 2009**

| Inclusion/exclusion criteria | Students living within Washington state at the time of the fall survey and that had parents who spoke English, Korean, or Vietnamese. |
| --- | --- |
| Objective | To explore time and efficiency of a web mode survey with additional in-person interviews and to examine whether sensitive questions (drug and sexual activity) differ by mode. |
| Trial date | The sample for this study was collected in 2004. |
| Data (participants) | Mean age: 18.6 years, range 17 to 19 years  Male (%): 58%  26% were living in low-income families at the beginning of the study  White (%): 82% on average  Asian or Pacific Islander (%): 8% on average  Hispanic (%): 4% on average  African American (%): 3% on average  Native American (%): 3% on average |
| Parents involved | NR |
| Comparisons | Mode 1: In-person first (n=189), students were first offered an in-person survey, with web follow-up.  Mode 2: Web first (n=197), students were asked to complete a survey over the internet and later offered the opportunity to complete the survey in person. |
| Strategy | Reminders, emails, and phone calls.  Advance letters were mailed to participants in both modes on their release date. The participants in the web-first mode received an email reminder six and fourteen days after the surveys release into the field, with telephone follow-up beginning twenty-five days after the release date. |
| Incentive | Throughout the study, small gifts (e.g., a clock radio) or monetary incentives were given to those who completed the survey (US$20 cash). |
| Survey(s) | Raising Healthy Children (RHC) Project: 14 items on substance use and sexual risk behavior |
| Field of study | Behaviour (sex risk behaviour, substance use, respondents’ social environment) |
| Recruitment strategy | Students enrolled in 1^st^ and 2^nd^ grade and followed up every spring. When participants turn 18 years of age, they provided consent for participation and these individuals formed the study cohort. |
| Data management, number of participants withdrawn or not responding | Missing data:  In-person first: 2.30 (SD 3.55)  Web first: Average 1.89 (SD 3.04)  Difference: *p* >.05 |
| Funding and Conflicts of interests | Supported by research grant # R01 DA08093-13 from the National Institute on Drug Abuse  Conflicts of interest: NR |

NR – not reported

**Comparison 5: Voucher vs no voucher**

**Pejtersen 2020**

| Inclusion/exclusion criteria | The included children came from families with severe social problems (e.g., foster care or former foster care youth, vulnerable families, lonely children and youths).  Participants not available in the Civil Register because of death, immigration or who had requested survey exemption were excluded. |
| --- | --- |
| Objective | To investigate if an unconditional monetary incentive can increase the response rate for vulnerable children and youths in a postal questionnaire survey. |
| Trial date | 2014 |
| Data (participants) | Mean age (SD):  Voucher 16.3 (0.68) years; non-voucher 17.1 (3.6) years, range: 11 to 28 years  Male (%): 32%  Institutionalized children and youth  Children in foster care or former foster care youths: voucher (35%); non-voucher (32%)  Children from vulnerable families: voucher (43%); non-voucher (34%)  Lonely children and youths: voucher (34%); non-voucher (26%) |
| Parents involved | For participants under 16 years of age, written informed consent was obtained from a parent. For all other participants, consent was given by the participants themselves. |
| Comparisons | Mode 1: Voucher (n=143)  Mode 2: Non-voucher (n=119) |
| Strategy | Postal survey sent to participants’ addresses. |
| Incentive | Intervention: supermarket voucher of €15 |
| Survey(s) | The Danish version of the Strengths and Difficulties Questionnaire (SDQ). |
| Field of study | Mental health and participants’ life situation (social skills and social relations initiative)  Surveys: Ad-hoc questionnaire on participants’ life situation: family and housing; education and training; sport and leisure time; relation to friends; drug use; and strengths and difficulties. |
| Recruitment strategy | Participants were recruited from an evaluation study of a social initiative aimed at creating networks for different groups of vulnerable children and youths aged 8–23 years. |
| Data management, number of participants withdrawn or not responding | 3 participants in the intervention group and 2 in the control were withdrawn because the letter could not be delivered to the address. |
| Funding and Conflicts of interests | The data were collected as part of a study funded by Danish National Board of Social Services. The trial was retrospectively registered at ClinicalTrials.gov (Identifier: NCT01741675)  Conflicts of interests: none |

SD – standard deviation

**Comparison 6: Internal supervision vs external supervision**

**Walser 2012**

| Inclusion/exclusion criteria | Public high school/private schools and schools for children with disabilities. |
| --- | --- |
| Objective | To look at the validity of sensitive data collected online when students are supervised by their teacher or, by an external person. |
| Trial date | 2008 |
| Data (participants) | 9th grade students, aged 15-16 years.  Males and females did not differ between groups. |
| Parents involved | Passive consent was applied, but there were no refusals to participate. |
| Comparisons | Mode 1. External supervision (n= 40 classes, 598 students), an external person (i.e., a senior student or researcher) supervised the students during online interviews.  Mode 2. Internal supervision (n= 40 classes, 599 students), students were supervised by a teacher. |
| Strategy | The survey was mandated by the Swiss Government (Department of Security & Justice and Department of Education) and carried out by the Institute of Criminology, University of Zurich. |
| Incentive | Computers and the Internet were available in all classes. To guarantee anonymity, supervisors were advised not to walk around or to watch the computer screens while students were filling out the questionnaire. |
| Survey(s) | Computer-assisted-self-interviewing (CASI) using a program from NETQ. |
| Field of study | Behaviours (delinquency, victimization, and substance use) |
| Recruitment strategy | NR |
| Data management, number of participants withdrawn or not responding | Missing values were calculated as non-response, and were equal among the two groups. |
| Funding and Conflicts of interests | NR |

NR- not reported
