# Supplementary material for: Methods, strategies, and incentives to increase response to mental health surveys among adolescents: a systematic review
Source: BMC Med Res Methodol. 2023 Nov 16;23:270. doi: 10.1186/s12874-023-02096-z (PMC10652438; doi:10.1186/s12874-023-02096-z)

**Appendix D: Risk of bias assessments**

A major concern with 2/3 cluster trials was biases arising from the randomization process and the timing of the identification and recruitment of individual participants in relation to the timing of randomisation. None of the trials reported a protocol or trial registry record raising concerns about selective reporting of the results and there may be outcome measurement bias in 2/3 trials.

Most (10/12) of the parallel design RCTs provided no information about randomization. We assumed that deviations from the intended intervention probably did not occur, and we judged this as low risk in 9/12 studies. For 7/12 studies there were concerns and in 2/12 studies there was high RoB over missing outcome data. Poorly reported measurement of the outcome domains raised concerns in 9/12 studies. We could access protocol information for one study (Pejtersen, 2020) raising concerns about selective reporting in the remaining 11 studies.

**Figure D.1 Risk of bias assessments for the cluster trials**


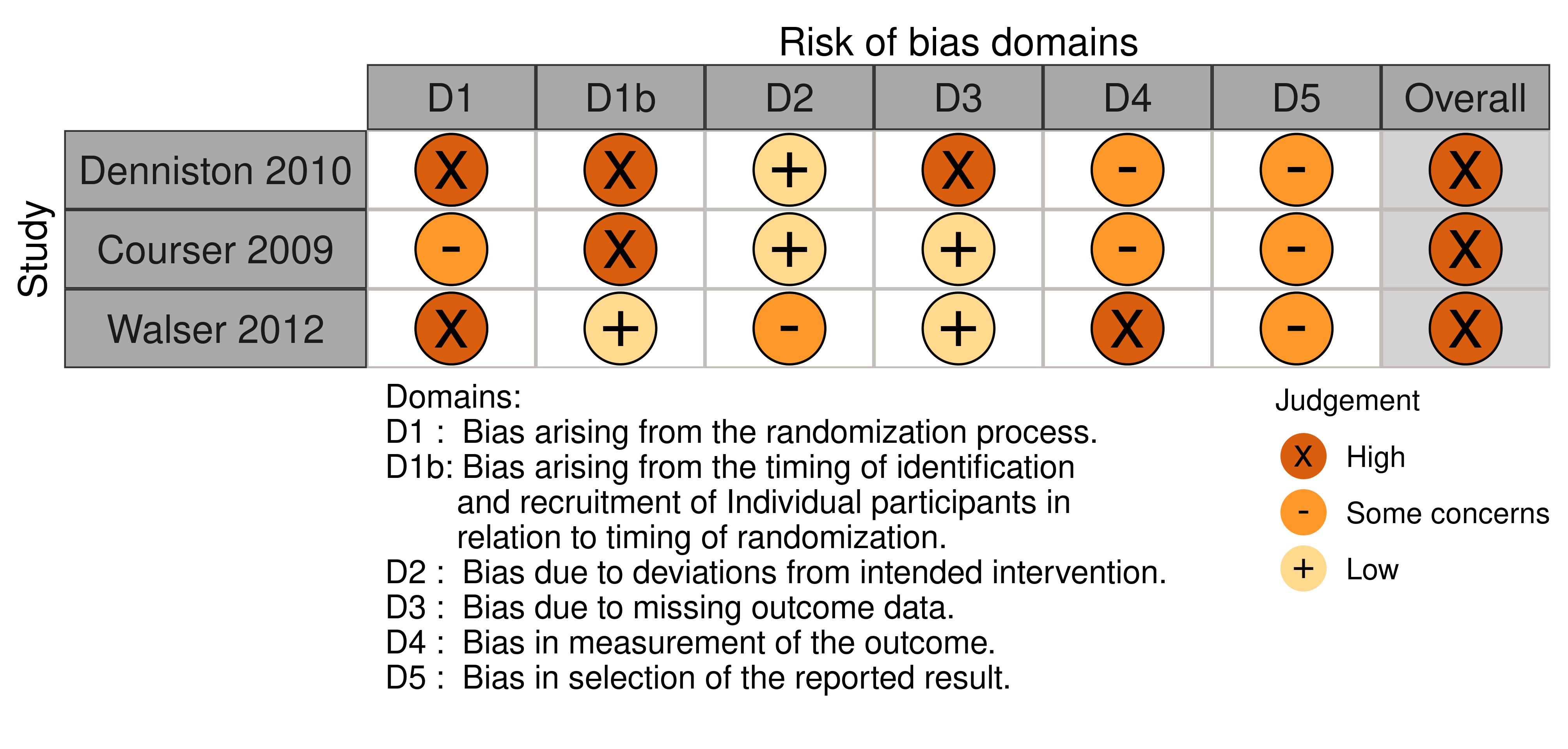


**Figure D.2 Risk of bias assessments for the randomized controlled trials**


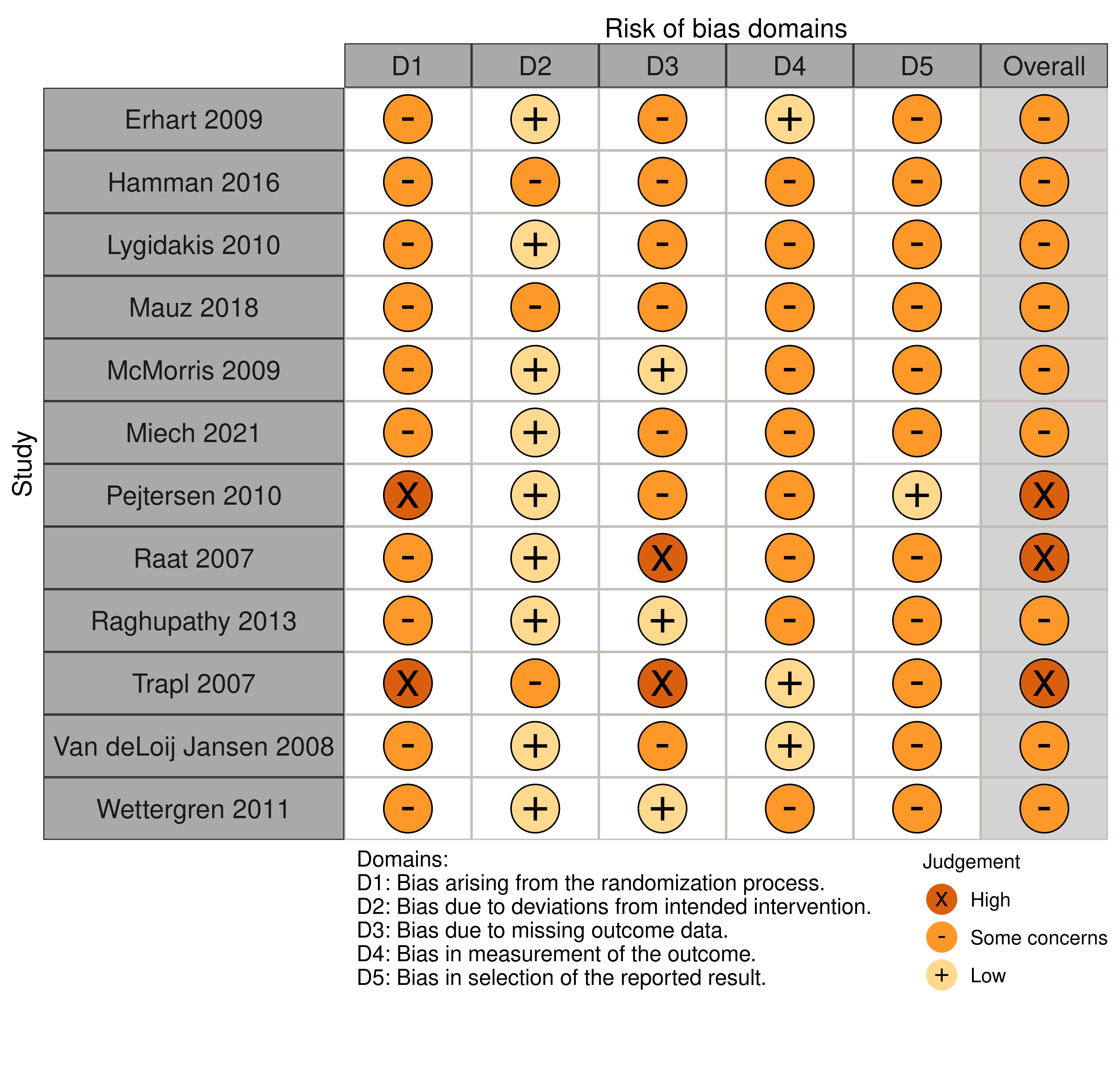

Supplement: Supplementary file 4 — Additional file 4. Risk of bias assessment. [file 12874_2023_2096_MOESM4_ESM.docx]
