# Supplementary material for: Methods, strategies, and incentives to increase response to mental health surveys among adolescents: a systematic review
Source: BMC Med Res Methodol. 2023 Nov 16;23:270. doi: 10.1186/s12874-023-02096-z (PMC10652438; doi:10.1186/s12874-023-02096-z)

**Appendix F: Additional Forest plots**

**Comparison 1: Paper-and-pencil versus web-based administration mode**

Figure F.1. Odds ratios for paper-and-pencil versus web-based survey delivery modes: Adolescents’ response rates (results not pooled)


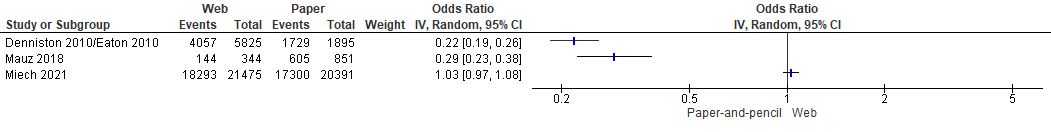


Sadness and suicide attempt

Figure F.2 Odds ratios for paper-and-pencil versus web-based survey administration: Sadness and suicide attempts among adolescents measured by the Youth Risk Behavior Surveys


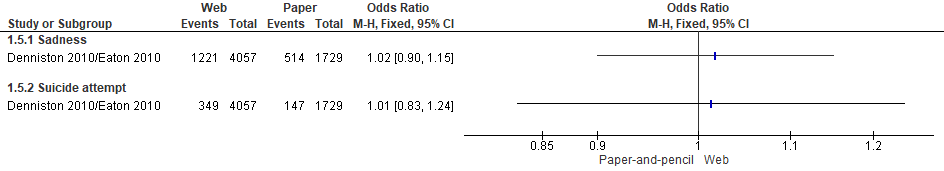


**Comparison 2: Telephone interview vs postal questionnaires**

Response rate

Figure F.3 Odds ratios for telephone interview versus postal questionnaire administration: adolecents’ response rates (results not pooled)


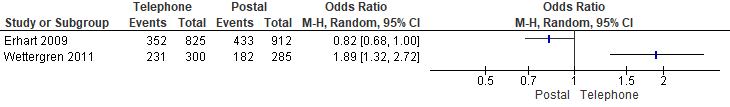


Estimate variations by mode of survey delivery: anxiety and depression

Figure F.4 Mean differences for telephone interview versus postal questionnaire delivery mode: adolescents’ self-reported anxiety and depression (results not pooled)


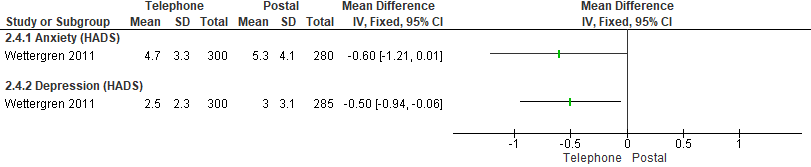


Note: The HADS is a fourteen-item scale that generates: Seven of the items relate to anxiety and seven relate to depression. Lower scores indicate better status ([link](https://www.svri.org/sites/default/files/attachments/2016-01-13/HADS.pdf)).

**Comparison 4: Web first vs in-person first survey versions**

Response rate

Figure F.5*.* Odds ratios for web version first compared with in-person first survey administration: adolescents’ response rates

**
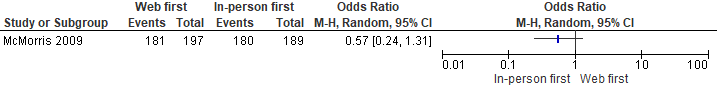
**

Estimate variations by mode of survey delivery: alcohol – lifetime use

Figure F.6 Odds ratios for web version first compared with in-person first survey administration: adolescents’ self-reported lifetime alcohol use

**
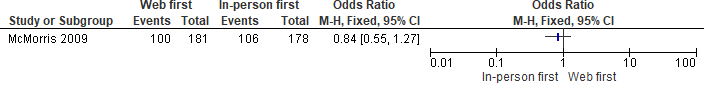
**

Estimate variations by mode of survey delivery: marijuana – lifetime use

Figure F.7 Odds ratios for web version first compared with in-person first survey administration: adolescents’ self-reported lifetime marijuana use

**
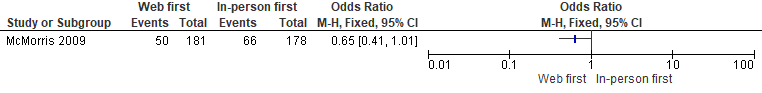
**

**Comparison 5: Voucher vs no voucher**

Response rate

Figure F.8 Odds ratios for surveys with a voucher versus no voucher: adolescents’ survey response rate


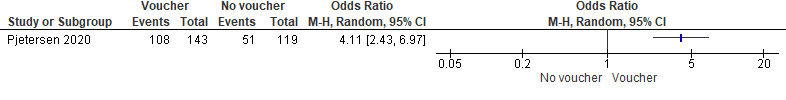

Estimate variations by mode of survey delivery: mental health (emotional symptoms)

Figure F.9 Mean difference for surveys with a voucher and no voucher: adolescents’ self-reported mental health


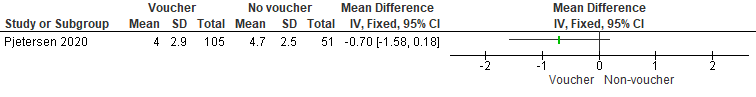


**Comparison 6: Internal versus external supervision**

Estimate variations by mode of survey delivery: mental health (emotional symptoms)

Figure F.10 Odds ratios for internal versus external supervision of online interviews: adolescents’ self-reported lifetime alcohol use


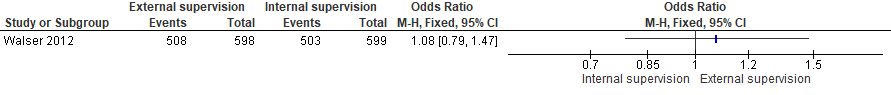

Supplement: Supplementary file 6 — Additional file 6. Additional Forest plots. [file 12874_2023_2096_MOESM6_ESM.docx]
