# Supplementary material for: Methods, strategies, and incentives to increase response to mental health surveys among adolescents: a systematic review
Source: BMC Med Res Methodol. 2023 Nov 16;23:270. doi: 10.1186/s12874-023-02096-z (PMC10652438; doi:10.1186/s12874-023-02096-z)
Supplement: Supplementary file 7 — Additional file 7. Protocol changes. [file 12874_2023_2096_MOESM7_ESM.docx]

Appendix F. Protocol changes

The protocol is publicly available (Bidonde 2022). This appendix lists the protocol changes made after the protocol had been finalised.

| Change | Reason for change |
| --- | --- |
| Eligibility criteria: outcomes | The wording of the two variations outcomes definitions were changed to make them clearer – the outcomes collected remain as intended. |
| We limited inclusion of studies to those conducted in high income countries |  |
| Changes to the naming of the eligible outcomes | The outcome labels were clarified... |
| GRADE assessment of the certainty of the evidence | The planned GRADE assessment did not prove feasible |
| Approaching authors for further information to fill gaps in the studies | We were unable to do this due to time constraints |
| Data analysis: dichotomous outcomes were planned to be presented as Risk ratios but this was changed to odds ratios |  |
